# Efficient Stereo-Selective Fluorination on Vitamin D_3_ Side-Chain Using Electrophilic Fluorination

**DOI:** 10.3390/biom14010037

**Published:** 2023-12-26

**Authors:** Fumihiro Kawagoe, Sayuri Mototani, Atsushi Kittaka

**Affiliations:** Faculty of Pharmaceutical Sciences, Teikyo University, 2-11-1 Kaga, Itabashi, Tokyo 173-8605, Japan; fkawagoe@pharm.teikyo-u.ac.jp (F.K.); 19dy10003vu@stu.teikyo-u.ac.jp (S.M.)

**Keywords:** electrophilic fluorination, *N*-fluorobenzenesulfonimide (NFSI), side-chain fluorinated vitamin D_3_, side-chain, Evans chiral auxiliary

## Abstract

Our research regarding side-chain fluorinated vitamin D_3_ analogues has explored a series of efficient fluorination methods. In this study, a new electrophilic stereo-selective fluorination methodology at C24 and C22 positions of the vitamin D_3_ side-chain was developed using *N*-fluorobenzenesulfonimide (NFSI) and CD-ring imides with an Evans chiral auxiliary (**26**,**27**,**30**).

## 1. Introduction

**Scheme 1:**
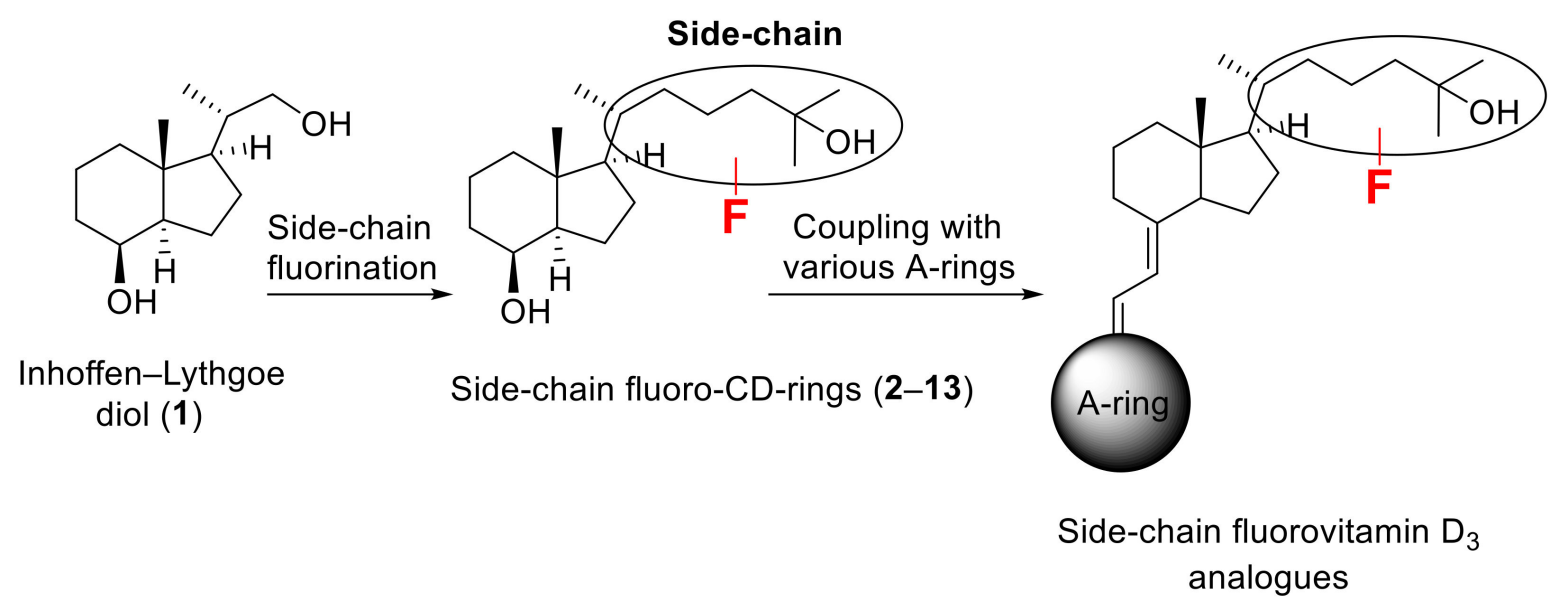
Convergent synthesis of side-chain fluorinated vitamin D_3_ analogues via side-chain fluoro-CD-ring fragments (**2–13**) starting from the Inhoffen–Lythgoe diol (**1**).

**Figure 1:**
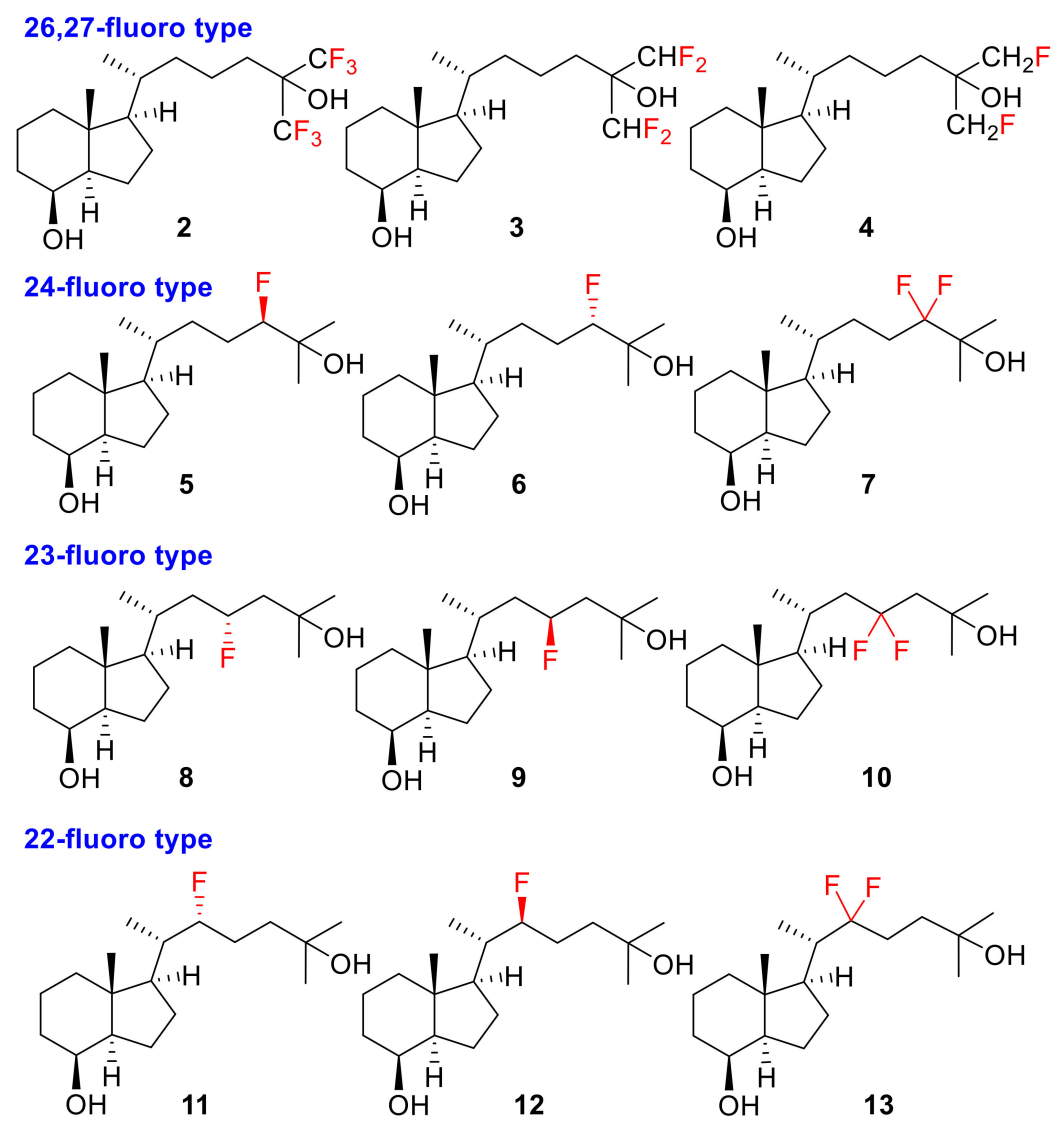
Structures of side-chain fluorinated CD-rings (**2–13**).

Regio- and stereo-selective fluorination of the vitamin D_3_ side-chain is one of the efficient methods used to modulate biological activities, such as binding affinity to the vitamin D receptor (VDR), transactivation activity through the VDR, and metabolic stability against CYP24A1 [1]. We have reported the regio- and stereo-selective fluorination of the CD-ring side-chain starting from the Inhoffen–Lythgoe diol (**1**) and constructed C26,27-fluoro-CD-rings (**2–4**) [2,3], C24-fluoro-CD-rings (**5–7**) [4,5], C23-fluoro-CD-rings (**8–10**) [6,7], and C22-fluoro-CD-rings (**11–13**) [8] (Figure 1) to create a chemical library of side-chain fluorovitamin D_3_ analogues using a convergent method (Scheme 1) [9,10,11].

### 1.1. Previous Studies of C24-Stereoselective Fluorination

On the vitamin D_3_ side-chain, the C24 position is an important site for metabolic inactivation via CYP24A1 hydroxylation [12,13,14]. Therefore, structural modifications, especially fluorination and difluorination, of the C24 position have been actively investigated [15,16,17,18,19,20,21,22,23,24,25].

**Scheme 2:**
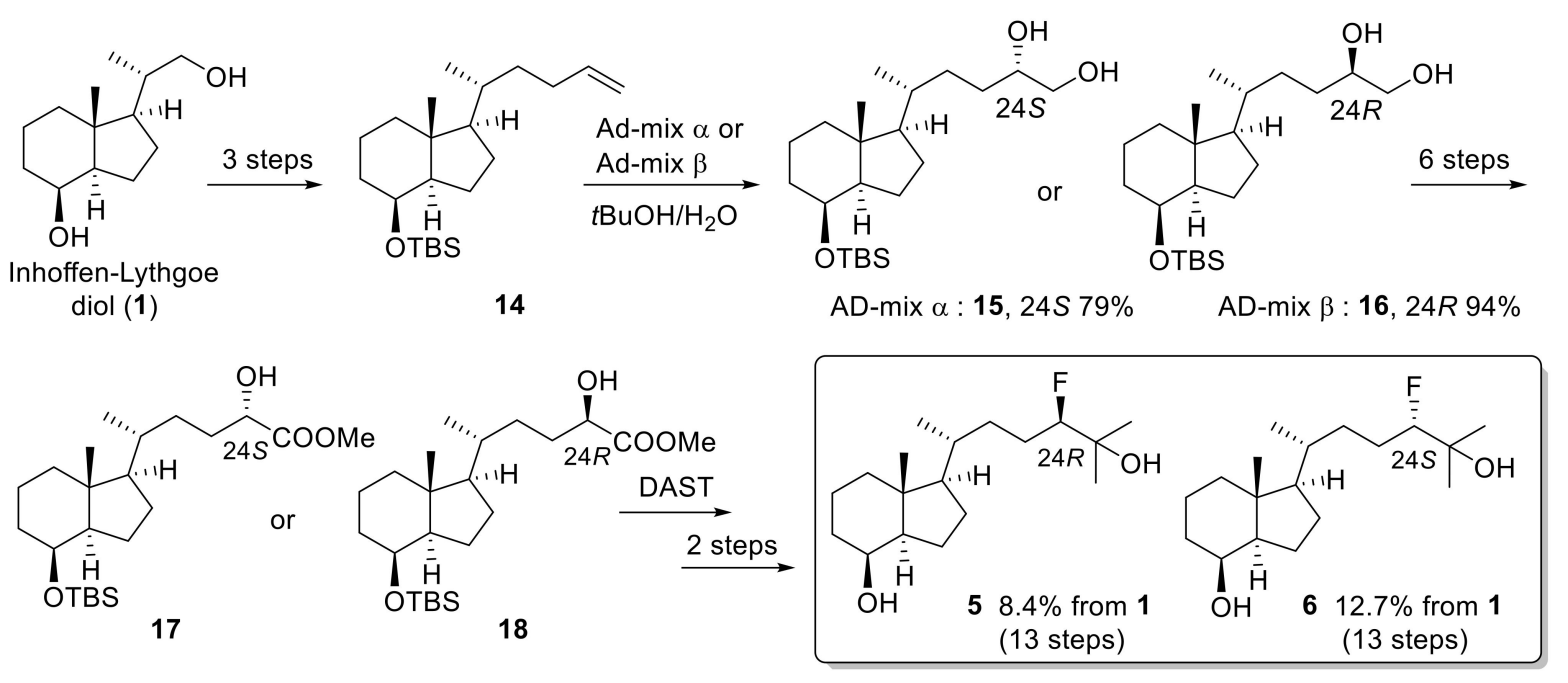
Synthetic route to C24-fluoro-CD-rings (**5**,**6**) using dihydroxylation/deoxyfluorination reactions.

We previously reported the stereo-selective fluorination method at the C24 position of the CD-ring side-chain for stereo-selective synthesis of (24*R*)-fluoro-25-hydroxyvitamin D_3_ and its 24*S* isomer [5]. The key step for introducing the C24-fluoro unit was achieved via osmium-catalyzed diastereoselective dihydroxylation followed by deoxy-fluorination reactions. However, multi-step synthesis was required (13 steps), and harmful osmium and excess amounts of the fluorinating reagent, DAST, were used in this synthetic route (Scheme 2).

### 1.2. Previous Studies of C22-Stereoselective Fluorination

**Scheme 3:**
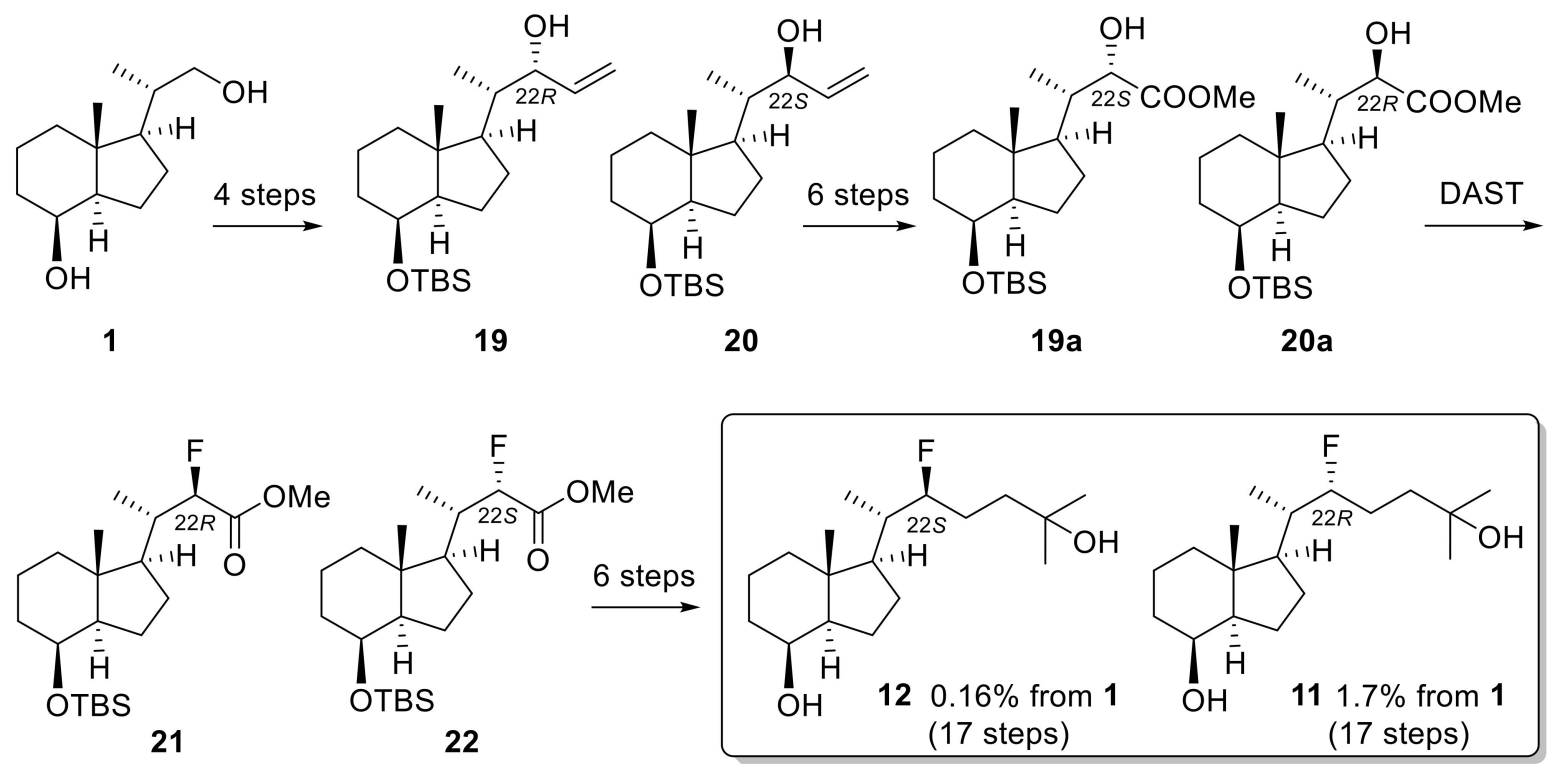
Preparation of C22-fluoro-CD-rings (**11**,**12**) using DAST for the deoxyfluorination step.

Until 2023, there was only one example of 22-fluorovitamin D synthesis, and the stereochemistry of C22 was unknown [26]. We recently reported the first stereo-selective fluorination at the C22 position of the CD-ring side-chain and synthesis of (22*R*)- and (22*S*)-fluoro-25-hydroxyvitamin D_3_ [8]. However, multiple synthetic steps were required, including separation of the diastereomer mixture of C22-allylalcohols (**19**,**20**) and the deoxyfluorination step using DAST, resulting in low overall yields (Scheme 3).

**Scheme 4:**
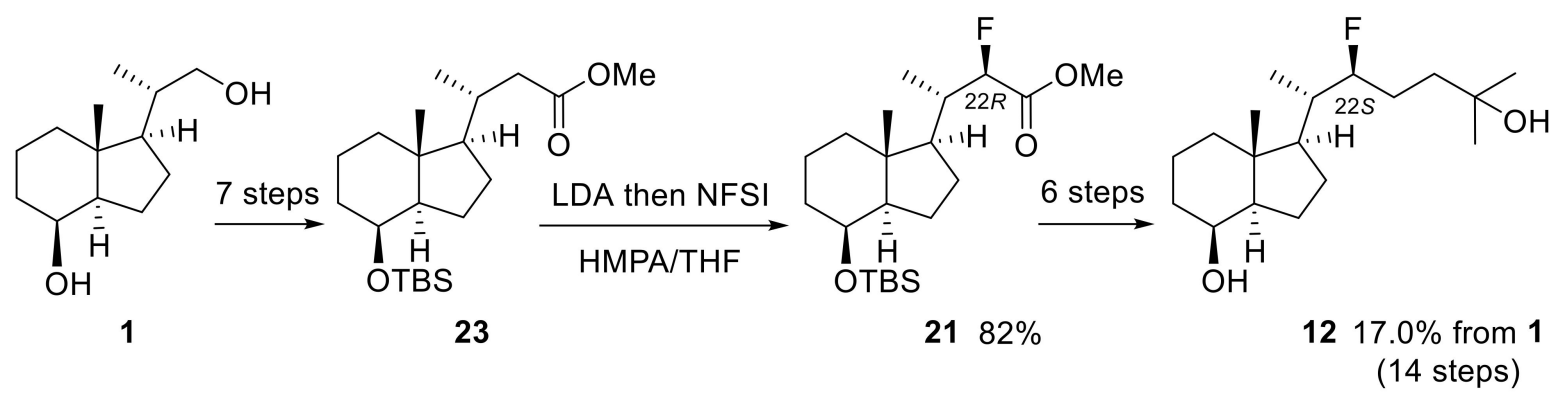
Electrophilic fluorination at the C22-position of the CD-ring side-chain.

In the report [8], we also developed an alternative C22-fluorination methodology using a cationic fluorination reaction between the methyl ester (**23**) and *N*-fluorobenzenesulfonimide (NFSI) under basic conditions. However, this route yielded only the (22*R*)-fluoro product (**21**); efficient cationic fluorination of the (22*S*)-fluoro counterpart to yield the (22*R*)-CD-ring (**11**) has yet to be explored (Scheme 4).

To overcome these drawbacks in order to introduce C24- and C22-monofluoro moieties, here we describe an alternative stereo-selective fluorination methodology at these positions via electrophilic fluorination using an Evans chiral auxiliary-based aldol reaction with NFSI [27,28,29].

**Scheme 5:**
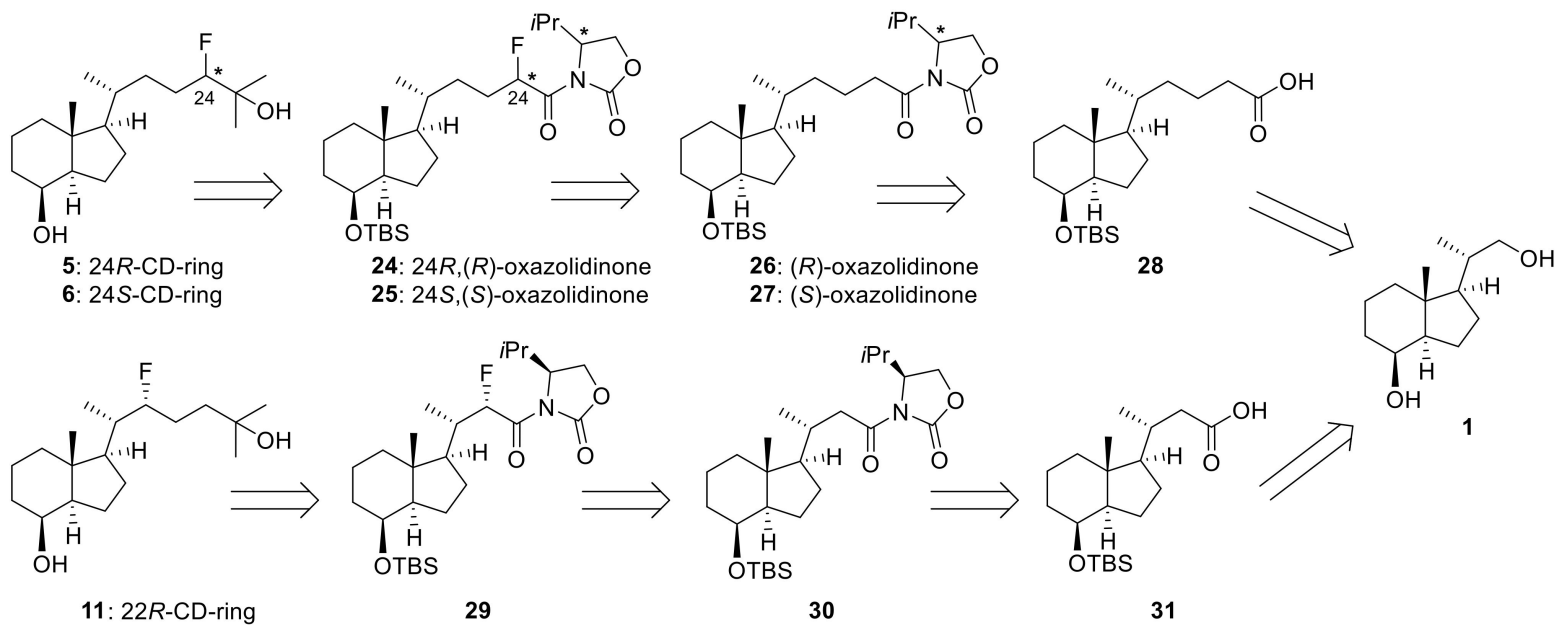
Synthetic plans for the C24- and C22-fluoro-CD-rings (**5**,**6**,**11**). * means a chiral center.

The retrosynthetic paths are shown in Scheme 5. Evans oxazolidinone ligands could be introduced to the side-chain carboxylic acids (**28**,**31**), which were synthesized from the Inhoffen–Lythgoe diol (**1**), and NFSI could be used as a cationic fluorination reagent in the presence of lithium hexamethyldisilazide (LHMDS).

## 2. Results and Discussion

### 2.1. Synthesis of 24-Fluoro-CD-Rings (**5**,**6**) via Electrophilic Fluorination

**Scheme 6:**
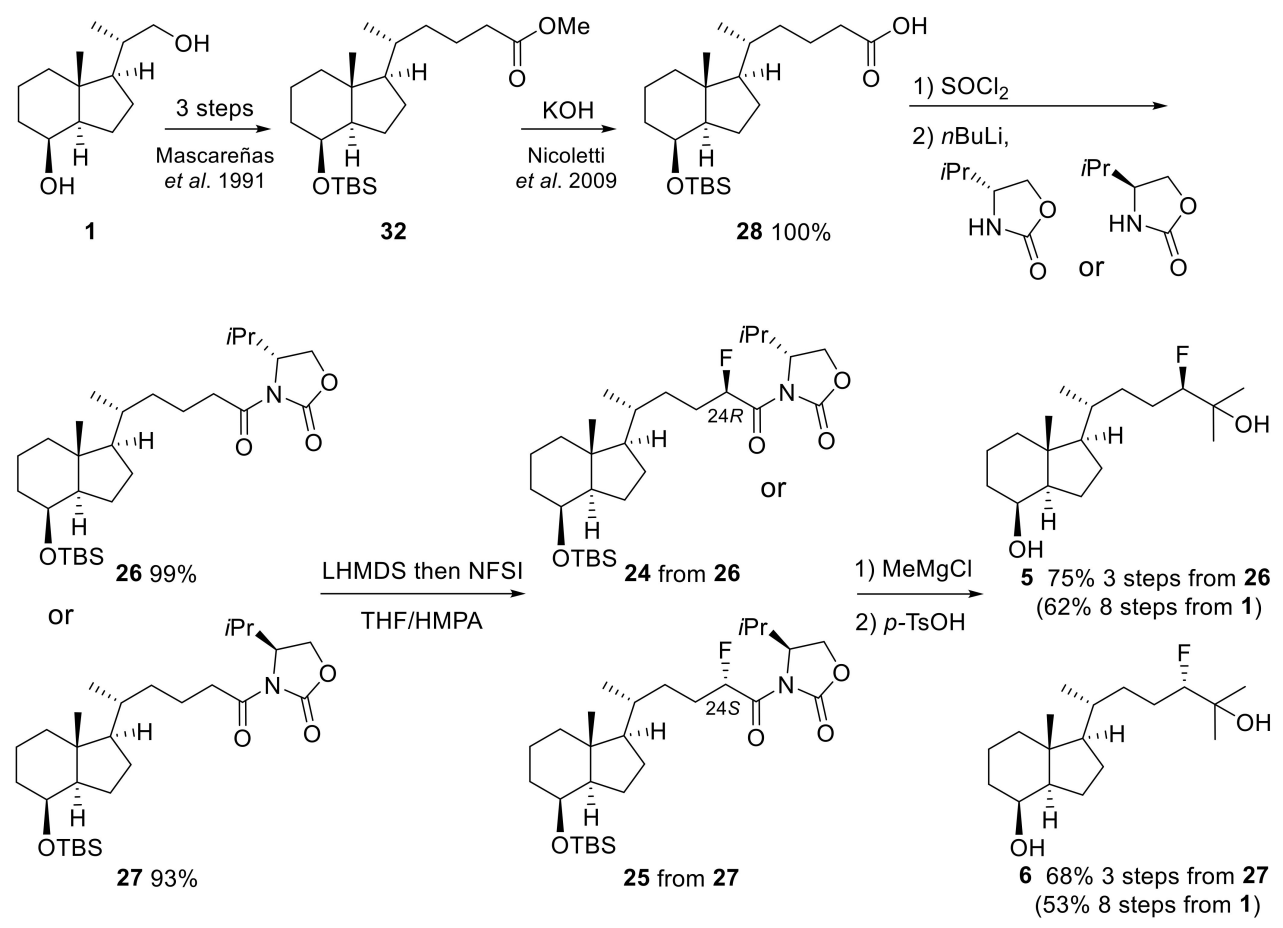
Improved synthesis of 24-fluoro-CD-rings (**5**,**6**) via electrophilic fluorination [30,31].

The synthetic route of the 24-fluoro-CD-rings (**5**,**6**) is illustrated in Scheme 6. We synthesized methyl ester (**32**) from the Inhoffen–Lythgoe diol (**1**) in three steps [30], and hydrolysis of **32** afforded the corresponding carboxylic acid (**28**) [31], which was subsequently converted to *N*-acyloxazolidinones (**26**,**27**) via acid chlorides. The C24-position was effectively fluorinated in a high diastereoselective manner to yield (24*R*)-product **24** from **26** and (24*S*)-product **25** from **27**, respectively. Displacement of the oxazolidinone ligand to 26,27-dimethyl units with the 25-hydroxy group using an excess amount of methyl magnesium chloride successfully proceeded with preservation of the stereochemistry at the C24-position, and removal of the silyl protective group under acidic conditions yielded the target (24*R*)-fluoro-CD-ring **5** (62% in 8 steps) and (24*S*)-fluoro-CD-ring **6** (53%).

### 2.2. Synthesis of (22R)-Fluoro-CD-Ring (**11**) via Electrophilic Fluorination

**Scheme 7:**
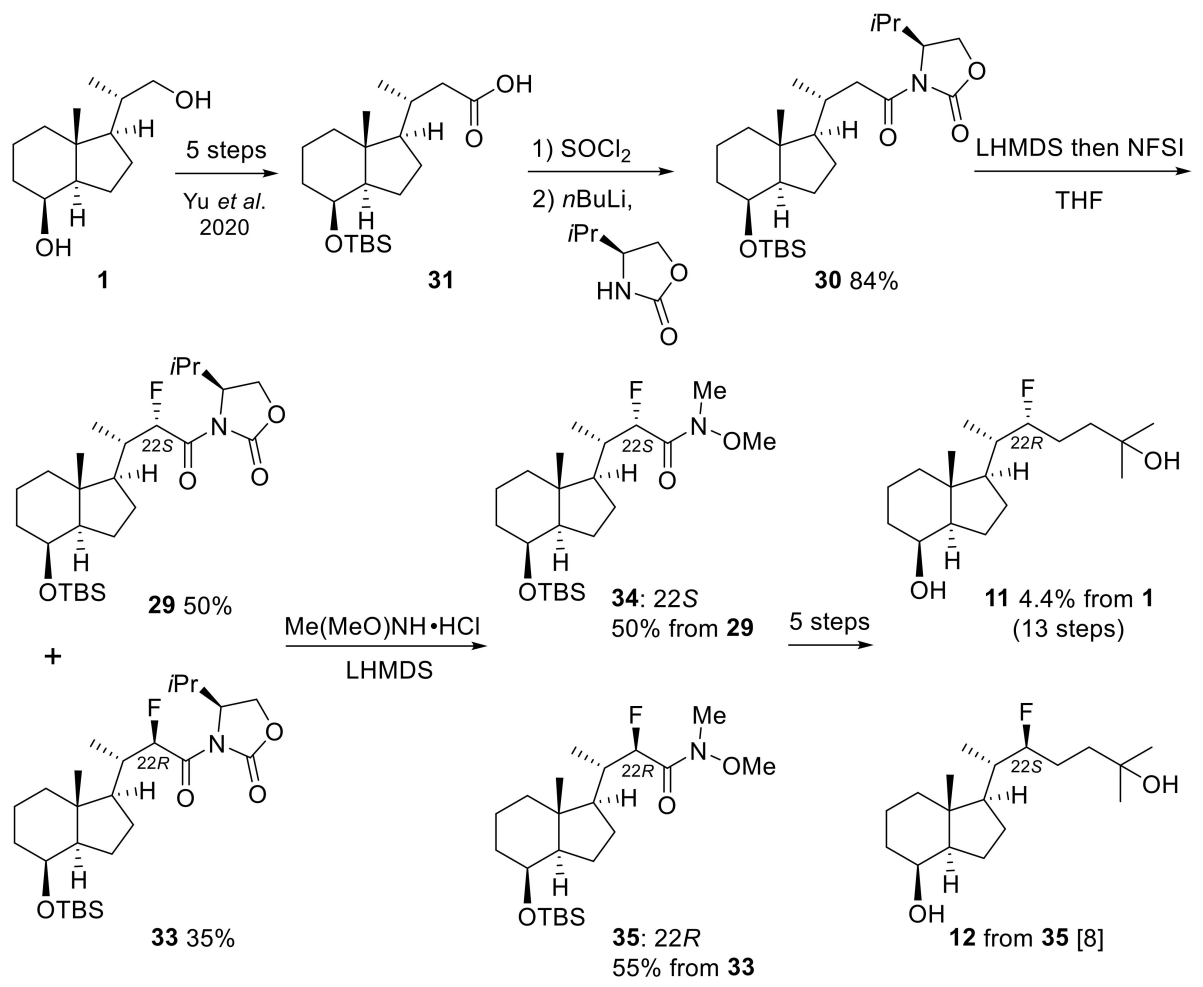
Improved synthesis of (22*R*)-fluoro-CD-ring (**11**) [32].

The synthetic route to the (22*R*)-fluoro-CD-ring (**11**) started from carboxylic acid (**31**), which was readily available from the Inhoffen–Lythgoe diol in five steps [32]. This was converted to amide (**30**) followed by fluorination at the C22-position using NFSI in the presence of LHMDS to give the desired (22*S*)-fluoro-CD-ring (**29**) as a major product, along with (22*R*)-fluoro-CD-ring (**33**) at a ratio of 1.4:1. After separating **29** and **33** using silica gel column chromatography, they were converted to the corresponding Weinreb amides (**34**,**35**), which were the synthetic intermediates of C22-fluoro-CD-rings **11** and **12** [8], using methoxy(methyl)amine hydrochloride and LHMDS in moderate yields and with a rigid stereochemical integrity (Scheme 7).

## 3. Conclusions

In conclusion, we developed an improved stereo-selective fluorination method at C24 and C22 positions to synthesize C24- and C22-fluoro-CD-rings utilizing an Evans chiral auxiliary for a cationic fluorination reaction as a key step. The side-chain carboxylic acids (**28**,**31**) were prepared from the Inhoffen–Lythgoe diol. These synthetic routes are more efficient than previous ones reported regarding both total yields and reaction steps.

**Experimental Section**

^1^H and ^13^C NMR spectra were recorded on JEOL AL-400 NMR (400 MHz) and ECP-600 NMR (600 MHz) spectrometers (Tokyo, Japan). ^1^H NMR spectra were referenced using (CH_3_)_4_Si (δ 0.00 ppm) or CHCl_3_ (δ 7.26 ppm) as an internal standard. ^13^C NMR spectra were referenced using deuterated solvent (δ 77.0 ppm for CDCl_3_). IR spectra were recorded using a JASCO FT-lR-800 Fourier transform infrared spectrophotometer (Tokyo, Japan). High-resolution mass spectra were obtained using a SHIMADZU LCMS-IT-TOF mass spectrometer (Kyoto, Japan) using a positive electrospray ionization (ESI) method. Optical rotations were measured using a JASCO DIP-370 digital polarimeter (Tokyo, Japan). Column chromatography was performed on silica gel 60N (Kanto Chemical Co., Inc., 40–50 μm, Tokyo, Japan) or silica gel 60 (Merck, 0.040–0.063 mm, Tokyo, Japan). All experiments were performed under anhydrous conditions in an atmosphere of argon, unless otherwise stated. Supporting information regarding ^1^H and ^13^C NMR spectra of all new compounds (**26**, **27**, **29**, **30**, and **33**) is available via the link in Supplementary Materials.

**(4*R*)-3-[(5*R*)-5-{(1*R*,3a*R*,4*S*,7a*R*)-4-[(*tert*-Butyldimethylsilyl)oxy]-7a-methyloctahydro-1*H*-inden-1-yl}hexanoyl]-4-isopropyloxazolidin-2-one (26)**

Carboxylic acid **28** [31] (403.6 mg, 1.05 mmol) was dissolved in thionyl chloride (3.0 mL, 4.97 g, 41.8 mmol) at room temperature, and the solution was refluxed for 1 h 30 min. The mixture was evaporated in vacuo, and the crude acyl chloride was used for the next reaction without further purification.

*n*BuLi (1.59 M in hexane, 5.3 mL, 8.40 mmol) was added to a precooled solution of (*R*)-4-isopropyloxazolidinone (1.09 g, 8.40 mmol) in THF (20 mL) at −78 °C, and the mixture was stirred at the same temperature for 10 min. The above acyl chloride in THF (3 mL) was added to the solution and stirred at the same temperature for 10 min. After the reaction was quenched with H_2_O and saturated aqueous NH_4_Cl at room temperature, the mixture was extracted with EtOAc three times. The organic layer was dried over Na_2_SO_4_, filtered, and concentrated. The residue was purified via flash column chromatography on silica gel (hexane:EtOAc = 3:1) to obtain **26** (513.6 mg, 99%, in 2 steps) as a colorless oil.

**26**: [α] _D_^27^ + 6.1 (c 2.01, CHCl_3_); IR (neat) 1786, 1704, 1383, 1252, 1208, 1085, 1023, 841 cm^−1^; ^1^H NMR (600 MHz, CDCl_3_) δ –0.02 (s, 3H), –0.01 (s, 3H), 0.86–0.91 (m, 21H), 1.02–1.12 (m, 3H), 1.18–1.25 (m, 2H), 1.29–1.44 (m, 5H), 1.47–1.56 (m, 2H), 1.64–1.66 (m, 1H), 1.69–1.83 (m, 3H), 1.92–1.95 (m, 1H), 2.34–2.39 (m, 1H), 2.80–2.95 (m, 2H), 3.97–3.99 (m, 1H), 4.18–4.27 (m, 2H), 4.42 (dt, *J* = 3.0, 8.4 Hz, 1H); ^13^C NMR (150 MHz, CDCl_3_) δ –5.2, –4.8, 13.7, 14.6, 17.6, 18.0, 18.0, 18.5, 21.0, 23.0, 25.8, 27.2, 28.4, 34.4, 35.1, 35.2, 35.9, 40.7, 42.1, 53.0, 56.5, 58.4, 63.3, 69.4, 154.0, 173.4; HRMS (ESI^+^) calcd for C_28_H_51_NO_4_SiNa [M+Na]^+^ 516.3480, found 516.3489.

**(4*S*)-3-[(5*R*)-5-{(1*R*,3a*R*,4*S*,7a*R*)-4-[(*tert*-Butyldimethylsilyl)oxy]-7a-methyloctahydro-1*H*-inden-1-yl}hexanoyl]-4-isopropyloxazolidin-2-one (27)**

Carboxylic acid **28** (606.2 mg, 1.58 mmol) was dissolved in thionyl chloride (3.4 mL, 5.6 g, 47.0 mmol) at room temperature, and the solution was refluxed for 1 h 30 min. The mixture was evaporated in vacuo, and the crude acyl chloride was used for the next reaction without further purification.

*n*BuLi (1.59 M in hexane, 5.9 mL, 9.42 mmol) was added to a precooled solution of (*S*)-4-isopropyloxazolidinone (1.24 g, 9.60 mmol) in THF (20 mL) at −78 °C, and the mixture was stirred at the same temperature for 10 min. The above acyl chloride in THF (4 mL) was added to the solution and stirred at the same temperature for 20 min. After the reaction was quenched with H_2_O and saturated aqueous NH_4_Cl at room temperature, the mixture was extracted with EtOAc three times. The organic layer was dried over Na_2_SO_4_, filtered, and concentrated. The residue was purified via flash column chromatography on silica gel (hexane:EtOAc = 3:1) to obtain **27** (729.5 mg, 93%, in 2 steps) as a colorless oil.

**27**: [α] _D_^27^ + 78.4 (c 1.58, CHCl_3_); IR (neat) 1786, 1704, 1387, 1255, 1208, 1089, 1027, 841 cm^−1^; ^1^H NMR (600 MHz, CDCl_3_) δ –0.02 (s, 3H), 0.00 (s, 3H), 0.86–0.92 (m, 21H), 1.00–1.11 (m, 3H), 1.19–1.25 (m, 2H), 1.29–1.44 (m, 5H), 1.49–1.57 (m, 2H), 1.61–1.83 (m, 4H), 1.92–1.95 (m, 1H), 2.33–2.40 (m, 1H), 2.76–2.99 (m, 2H), 3.97–3.99 (m, 1H), 4.18–4.27 (m, 2H), 4.42 (dt, *J* = 3.3, 8.4 Hz, 1H); ^13^C NMR (150 MHz, CDCl_3_) δ –5.2, –4.8, 13.7, 14.6, 17.6, 18.0, 18.0, 18.5, 21.0, 23.0, 25.8, 27.2, 28.4, 34.4, 35.1, 35.2, 36.0, 40.7, 42.1, 53.0, 56.5, 58.3, 63.3, 69.4, 154.1, 173.5; HRMS (ESI^+^) calcd for C_28_H_51_NO_4_SiNa [M+Na]^+^ 516.3480, found 516.3495.

**(1*R*,3a*R*,4*S*,7a*R*)-1-[(2*R*,5*R*)-5-Fluoro-6-hydroxy-6-methylheptan-2-yl]-7a-methyloctahydro-1*H*-inden-4-ol (5)**

To a solution of **26** (168.9 mg, 0.34 mmol) in THF (2 mL) and hexamethylphosphoric triamide (HMPA) (200 μL) was added LHMDS (lithium hexamethyldisilazide) (359 μL, 1 M THF solution, 0.36 mmol) at −78 °C, the mixture was stirred at the same temperature for 10 min, and *N*-fluorobenzenesulfonimide (NFSI) (117.8 mg, 0.37 mmol) was added. After being stirred at the same temperature for 40 min, the reaction mixture was quenched with H_2_O and saturated aqueous NH_4_Cl at −78 °C. The mixture was extracted with EtOAc three times. The organic layer was washed with brine, dried over Na_2_SO_4_, filtered, and concentrated. Crude **24** was used for the next reaction without further purification.

To a solution of the above crude **24** in THF (10 mL) was added MeMgCl (1.14 mL, 3.0 M THF solution, 3.42 mmol) at 0 °C, and the mixture was stirred at 0 °C for 10 min. After the reaction was quenched with H_2_O, the mixture was extracted with EtOAc three times. The organic layer was washed with saturated aqueous NH_4_Cl, dried over Na_2_SO_4_, filtered, and concentrated. The crude residue was used for the next reaction without further purification.

To the above crude residue in MeOH (15 mL) was added *p*-toluenesulfonic acid monohydrate (479.9 mg, 2.52 mmol), and the mixture was stirred at room temperature for 63 h under air. After the reaction was quenched with H_2_O and saturated aqueous NaHCO_3_ at room temperature, the mixture was extracted with EtOAc three times. The organic layer was dried over Na_2_SO_4_, filtered, and concentrated. The residue was purified via flash column chromatography on silica gel (hexane:EtOAc = 2:1) to obtain **5** (77.5 mg, 75%, in 3 steps) as a white powder. The spectral data matched with those reported in the literature [5].

**(1*R*,3a*R*,4*S*,7a*R*)-1-[(2*R*,5*S*)-5-Fluoro-6-hydroxy-6-methylheptan-2-yl]-7a-methyloctahydro-1*H*-inden-4-ol (6)**

To a solution of **27** (111.0 mg, 0.23 mmol) in THF (2 mL) and hexamethylphosphoric triamide (HMPA) (200 μL) was added LHMDS (lithium hexamethyldisilazide) (236 μL, 1 M THF solution, 0.24 mmol) at −78 °C, the mixture was stirred at the same temperature for 10 min, and *N*-fluorobenzenesulfonimide (NFSI) (76.4 mg, 0.24 mmol) was added. After being stirred at the same temperature for 40 min, the reaction mixture was quenched with H_2_O and saturated aqueous NH_4_Cl at −78 °C. The mixture was extracted with EtOAc three times. The organic layer was washed with brine, dried over Na_2_SO_4_, filtered, and concentrated. Crude **25** was used for the next reaction without further purification.

To a solution of the above crude **25** in THF (7 mL) was added MeMgCl (0.75 mL, 3.0 M THF solution, 2.25 mmol) at 0 °C, and the mixture was stirred at 0 °C for 10 min. After the reaction was quenched with H_2_O, the mixture was extracted with EtOAc three times. The organic layer was washed with saturated aqueous NH_4_Cl, dried over Na_2_SO_4_, filtered, and concentrated. The crude residue was used for the next reaction without further purification.

To the above crude residue in MeOH (10 mL) was added *p*-toluenesulfonic acid monohydrate (558.3 mg, 2.94 mmol), and the mixture was stirred at room temperature for 18 h under air. After the reaction was quenched with H_2_O and saturated aqueous NaHCO_3_ at room temperature, the mixture was extracted with EtOAc three times. The organic layer was dried over Na_2_SO_4_, filtered, and concentrated. The residue was purified via flash column chromatography on silica gel (hexane:EtOAc = 2:1) to obtain **6** (45.9 mg, 68%, in 3 steps) as a white powder. The spectral data matched with those reported in the literature [5].

**(4*S*)-3-[(3*R*)-3-{(1*R*,3a*R*,4*S*,7a*R*)-4-[(*tert*-Butyldimethylsilyl)oxy]-7a-methyloctahydro-1*H*-inden-1-yl}butanoyl]-4-isopropyloxazolidin-2-one (30)**

Carboxylic acid **31** [32] (613.8 mg, 1.73 mmol) was dissolved in thionyl chloride (2.5 mL, 4.12 g, 34.6 mmol) at room temperature, and the solution was refluxed for 1 h 30 min. The mixture was evaporated in vacuo, and the crude acyl chloride was used for the next reaction without further purification.

*n*BuLi (1.59 M in hexane, 3.3 mL, 5.19 mmol) was added to a precooled solution of (*S*)-4-isopropyloxazolidinone (673.3 mg, 5.21 mmol) in THF (6 mL) at −78 °C, and the mixture was stirred at the same temperature for 15 min. The above acyl chloride in THF (6 mL) was added to the solution and stirred at the same temperature for 15 min. After the reaction was quenched with H_2_O and saturated aqueous NH_4_Cl at room temperature, the mixture was extracted with EtOAc three times. The organic layer was dried over Na_2_SO_4_, filtered, and concentrated. The residue was purified via flash column chromatography on silica gel (hexane:EtOAc = 4:1) to obtain **30** (679.3 mg, 84%, in 2 steps) as a colorless oil.

**30**: [α] _D_^27^ +67.9 (c 1.28, CHCl_3_); IR (neat) 1778, 1700, 1471, 1383, 1259, 1212, 1104, 1019, 960, 849, 779 cm^−1^; ^1^H NMR (600 MHz, CDCl_3_) δ –0.02 (s, 3H), 0.00 (s, 3H), 0.86 (d, *J* = 6.6 Hz, 3H), 0.88 (s, 9H), 0.91 (d, *J* = 6.6 Hz, 3H), 0.94 (d, *J* = 6.6 Hz, 3H), 0.95 (s, 3H), 1.10–1.17 (m, 2H), 1.23–1.27 (m, 1H), 1.32–1.43 (m, 4H), 1.53–1.61 (m, 1H), 1.64–1.68 (m, 1H), 1.76–1.84 (m, 2H), 1.94 (dt, *J* = 3.0, 12.6 Hz, 1H), 2.03–2.10 (m, 1H), 2.32–2.40 (m, 1H), 2.58 (dd, *J* = 9.6, 16.2 Hz, 1H), 2.58 (dd, *J* = 3.6, 15.6 Hz, 1H), 3.99–4.00 (m, 1H), 4.19 (dd, *J* = 3.3, 9.3 Hz, 1H), 4.25 (t, *J* = 8.7 Hz, 1H), 4.45 (dt, *J* = 3.6, 8.4 Hz, 1H); ^13^C NMR (150 MHz, CDCl_3_) δ –5.2, 4.8, 13.8, 14.5, 17.6, 18.0, 19.3, 23.0, 25.8, 27.0, 28.3, 32.6, 23.4, 40.6, 42.0, 42.3, 53.0, 56.9, 58.3, 63.1, 69.4, 154.0, 173.2; HRMS (ESI^+^) calcd for C_26_H_47_NO_4_SiNa [M+Na]^+^ 488.3167, found 488.3183.

**(4*S*)-3-[(2S,3*S*)-3-{(1*R*,3a*R*,4*S*,7a*R*)-4-[(*tert*-Butyldimethylsilyl)oxy]-7a-methyloctahydro-1*H*-inden-1-yl}-2-fluorobutanoyl]-4-isopropyloxazolidin-2-one (29) and (4*S*)-3-[(2*R*,3*S*)-3-{(1*R*,3a*R*,4*S*,7a*R*)-4-[(*tert*-Butyldimethylsilyl)oxy]-7a-methyloctahydro-1*H*-inden-1-yl}-2-fluorobutanoyl]-4-isopropyloxazolidin-2-one (33)**

To a solution of **30** (207.6 mg, 0.45 mmol) in THF (3 mL) was added LHMDS (lithium hexamethyldisilazide) (472 μL, 1 M THF solution, 0.472 mmol) at −78 °C, the mixture was stirred at 0 °C for 1 h, and *N*-fluorobenzenesulfonimide (NFSI) (168.6 mg, 0.53 mmol) was added to the mixture at −78 °C. After being stirred at the same temperature for 2 h, the reaction was quenched with H_2_O and saturated aqueous NH_4_Cl at −78 °C. The mixture was extracted with EtOAc three times. The organic layer was washed with brine, dried over Na_2_SO_4_, filtered, and concentrated. The residue was purified via flash column chromatography on silica gel (hexane:EtOAc = 2:1) to obtain **29** (108.4 mg, 50%) (less polar) and **33** (74.9 mg, 35%) (more polar), each as a colorless oil.

**29**: [α] _D_^27^ + 83.1 (c 1.08, CHCl_3_); IR (neat) 1786, 1718, 1384, 1256, 1201, 1025, 834 cm^−1^; ^1^H NMR (600 MHz, CDCl_3_) δ –0.02 (s, 3H), 0.00 (s, 3H), 0.88 (s, 9H), 0.89 (d, *J* = 7.2 Hz, 3H), 0.92 (s, 3H), 0.94 (d, *J* = 6.6 Hz, 3H), 1.14–1.39 (m, 9H), 1.52–1.67 (m, 3H), 1.74–1.94 (m, 3H), 2.08–2.18 (m, 1H), 2.51–2.56 (m, 1H), 3.98–4.00 (m, 1H), 4.29 (dd, *J* = 1.8, 9.0 Hz, 1H), 4.35 (t, *J* = 8.7 Hz, 1H), 4.38–4.40 (m, 1H), 5.74 (dd, *J* =2.7, 47.4 Hz, 1H); ^13^C NMR (150 MHz, CDCl_3_) δ –5.2, –4.8, 13.3, 14.4, 17.2 (d, *J* = 2.9 Hz), 17.5, 18.0, 18.0, 23.5, 25.8, 26.9, 28.0, 34.3, 40.1, 40.2, 40.3, 42.9, 51.6, 52.3, 59.1, 64.0, 69.3, 93.8 (d, *J* = 180.9 Hz), 153.5, 170.1 (d, *J* = 23.1 Hz); HRMS (ESI^+^) calcd for C_26_H_46_NO_4_FSiNa [M+Na]^+^ 506.3072, found 506.3075.

**33**: [α] _D_^27^ +1.2 (c 0.57, CHCl_3_); IR (neat) 1790, 1718, 1391, 1260, 1204, 1097, 1025, 978, 842, 774 cm^−1^; ^1^H NMR (600 MHz, CDCl_3_) δ –0.01 (s, 3H), 0.01 (s, 3H), 0.87 (d, *J* = 7.2 Hz, 3H), 0.88 (s, 9H), 0.92–0.95 (m, 9H), 1.18 (td, *J* = 2.4, 12.6 Hz, 1H), 1.32–1.43 (m, 4H), 1.49–1.68 (m, 4H), 1.76–1.93 (m, 4H), 2.03–2.17 (m, 1H), 2.30–2.37 (m, 1H), 4.00–4.02 (m, 1H), 4.26 (dd, *J* = 3.6, 9.0 Hz, 1H), 4.37 (t, *J* = 9.0 Hz, 1H), 4.57 (dt, *J* = 3.9, 9.0 Hz, 1H), 5.96 (dd, *J* = 1.5, 49.2 Hz, 1H); ^13^C NMR (150 MHz, CDCl_3_) δ –5.2, –4.9, 12.4 (d, *J* = 4.4 Hz), 13.7, 14.3, 17.6, 17.7, 17.9, 22.7, 25.0, 25.8, 28.0, 34.2, 37.1, 37.2, 40.6, 42.0, 52.7, 52.9, 57.7, 64.0, 69.3, 91.9 (d, *J* = 182.4 Hz), 153.4, 169.7 (d, *J* = 23.0 Hz); HRMS (ESI^+^) calcd for C_26_H_46_NO_4_FSiNa [M+Na]^+^ 506.3072, found 506.3091.

**(2*S*,3*S*)-3-{(1*R*,3a*R*,4*S*,7a*R*)-4-[(*tert*-Butyldimethylsilyl)oxy]-7a-methyloctahydro-1*H*-inden-1-yl}-2-fluoro-*N*-methoxy-*N*-methylbutanamide (34)**

To a solution of **29** (105.9 mg, 0.22 mmol) and Me(MeO)NH·HCl (48.8 mg, 0.50 mmol) in THF (10 mL) was added LHMDS (972 μL, 1 M in THF, 0.972 mmol) at 0 °C, and the mixture was stirred at the same temperature for 25 min. After the reaction was quenched with water and aqueous saturated NH_4_Cl, the mixture was extracted with EtOAc three times. The organic layer was dried over Na_2_SO_4_, filtered, and concentrated. The residue was purified via flash column chromatography on silica gel (hexane:EtOAc = 4:1) to obtain **34** (45.4 mg, 50%) as a colorless oil. The spectral data matched with those reported in the literature [8].

**(2*R*,3*S*)-3-{(1*R*,3a*R*,4*S*,7a*R*)-4-[(*tert*-Butyldimethylsilyl)oxy]-7a-methyloctahydro-1*H*-inden-1-yl}-2-fluoro-*N*-methoxy-*N*-methylbutanamide (35)**

To a solution of **33** (39.4 mg, 0.081 mmol) and Me(MeO)NH·HCl (17.2 mg, 0.18 mmol) in THF (5 mL) was added LHMDS (324 μL, 1 M in THF, 0.324 mmol) at 0 °C, and the mixture was stirred at the same temperature for 4 min. After the reaction was quenched with water and aqueous saturated NH_4_Cl, the mixture was extracted with EtOAc three times. The organic layer was dried over Na_2_SO_4_, filtered, and concentrated. The residue was purified via flash column chromatography on silica gel (hexane:EtOAc = 4:1) to obtain **35** (18.7 mg, 55%) as a colorless oil. The spectral data matched with those reported in the literature [8].

## Data Availability

The data presented in this study are available on request from the corresponding author.

## Supplementary Materials

The following supporting information can be downloaded at: https://www.mdpi.com/article/10.3390/biom14010037/s1. File S1: ^1^H and ^13^C NMR spectra of all new compounds **26**, **27**, **29**, **30**, and **33**.
