# Supplementary material for: Efficient Stereo-Selective Fluorination on Vitamin D3 Side-Chain Using Electrophilic Fluorination
Source: Biomolecules. 2023 Dec 26;14(1):37. doi: 10.3390/biom14010037 (PMC10812995; doi:10.3390/biom14010037)
Supplement: Supplementary file 1 [file biomolecules-14-00037-s001.zip › biomolecules-2785419-supplementary.pdf]

Supplementary Materials

Efficient Stereo-Selective Fluorination on Vitamin D_3 Side-Chain Using Electrophilic Fluorination

Fumihiro Kawagoe, Sayuri Mototani and Atsushi Kittaka *

Faculty of Pharmaceutical Sciences, Teikyo University, 2-11-1 Kaga, Itabashi, Tokyo 173-8605, Japan; fkawagoe@pharm.teikyo-u.ac.jp (F.K.); 19dy10003vu@stu.teikyo-u.ac.jp (S.M.)
* Correspondence: akittaka@pharm.teikyo-u.ac.jp; Tel.: +81-3-3964-8109; Fax: +81-3-3964-8117

Contents

Title page S1

^{1}H and ^{13}C NMR spectra of all new compounds **26**, **27**, **29**, **30**, and **33**. S2-S6

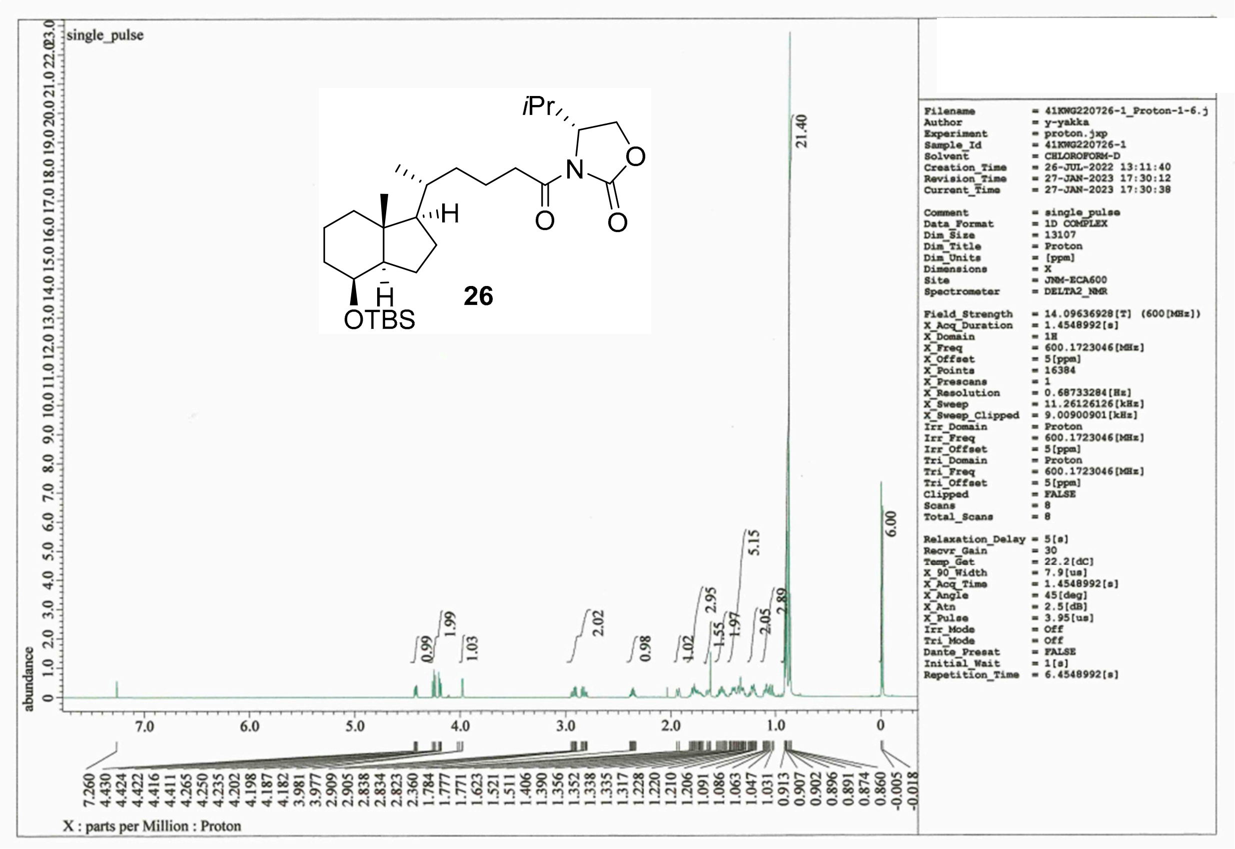
single_pulse
iPr
O
N
O
O
H
H
OTBS
26
21.40
6.00
5.15
2.95
2.89
2.05
1.97
1.55
1.02
0.98
2.02
1.03
1.99
0.99
abundance
X : parts per Million : Proton
Filename = 41KWG220726-1_Proton-1-6.j
Author = y-yakka
Experiment = proton.jxp
Sample_Id = 41KWG220726-1
Solvent = CHLOROFORM-D
Creation_Time = 26-JUL-2022 13:11:40
Revision_Time = 27-JAN-2023 17:30:12
Current_Time = 27-JAN-2023 17:30:38
Spectrometer = DELTA2_NMR
Field_Strength = 14.09636928[T] (600[MHz])
X_Domain = 1H
Scans = 8
Relaxation_Delay = 5[s]
Temp_Get = 22.2[dC]

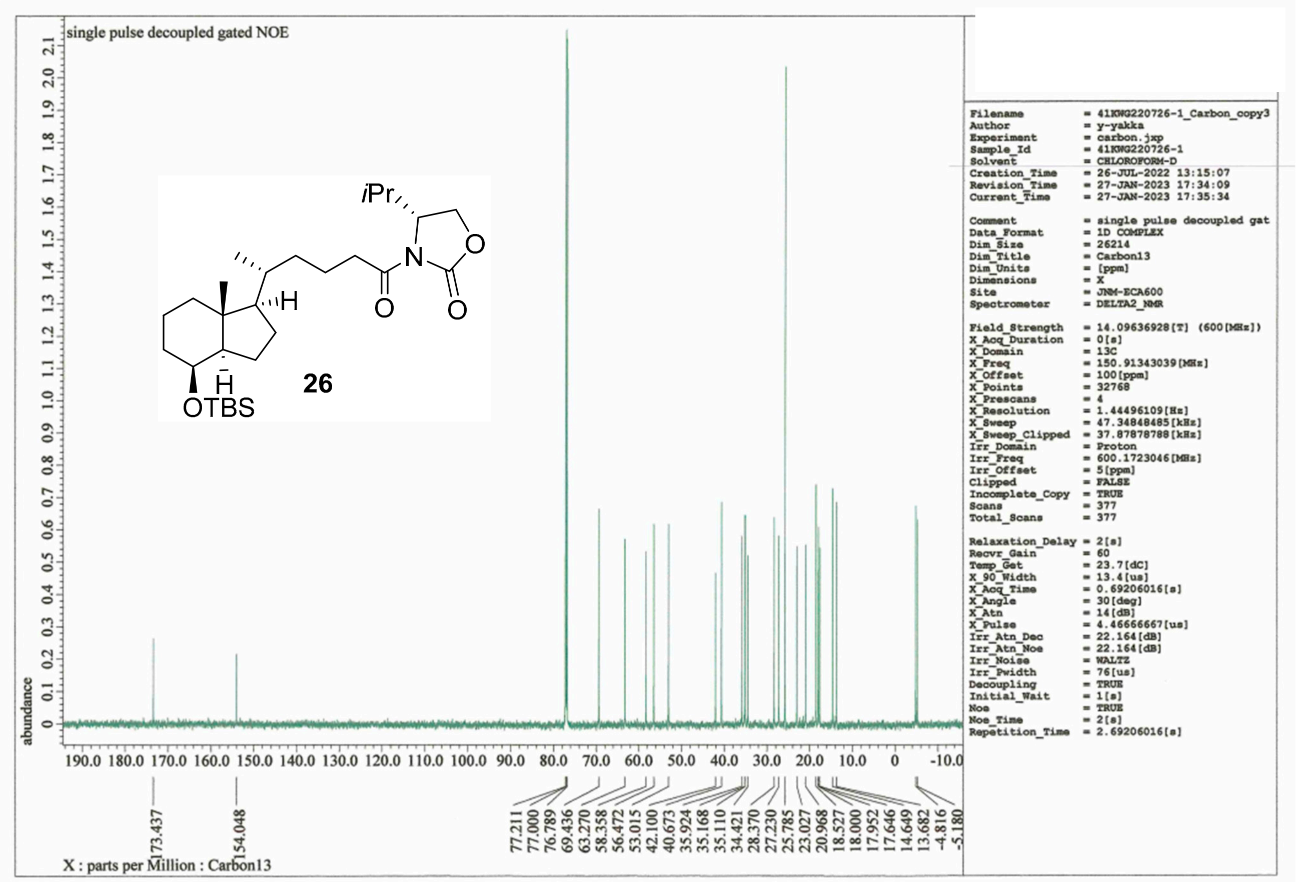
single pulse decoupled gated NOE
iPr
O
N
O
O
H
H
OTBS
26
abundance
X : parts per Million : Carbon13
173.437
154.048
77.211
77.000
76.789
69.436
63.270
58.358
56.472
53.015
42.100
40.673
35.924
35.168
35.110
34.421
28.370
27.230
25.785
23.027
20.968
18.527
18.000
17.952
17.646
14.649
13.682
-4.816
-5.180
Filename = 41KWG220726-1_Carbon_copy3
Author = y-yakka
Experiment = carbon.jxp
Sample_Id = 41KWG220726-1
Solvent = CHLOROFORM-D
Creation_Time = 26-JUL-2022 13:15:07
Revision_Time = 27-JAN-2023 17:34:09
Current_Time = 27-JAN-2023 17:35:34
Spectrometer = DELTA2_NMR
Field_Strength = 14.09636928[T] (600[MHz])
X_Domain = 13C
Scans = 377
Relaxation_Delay = 2[s]
Temp_Get = 23.7[dC]

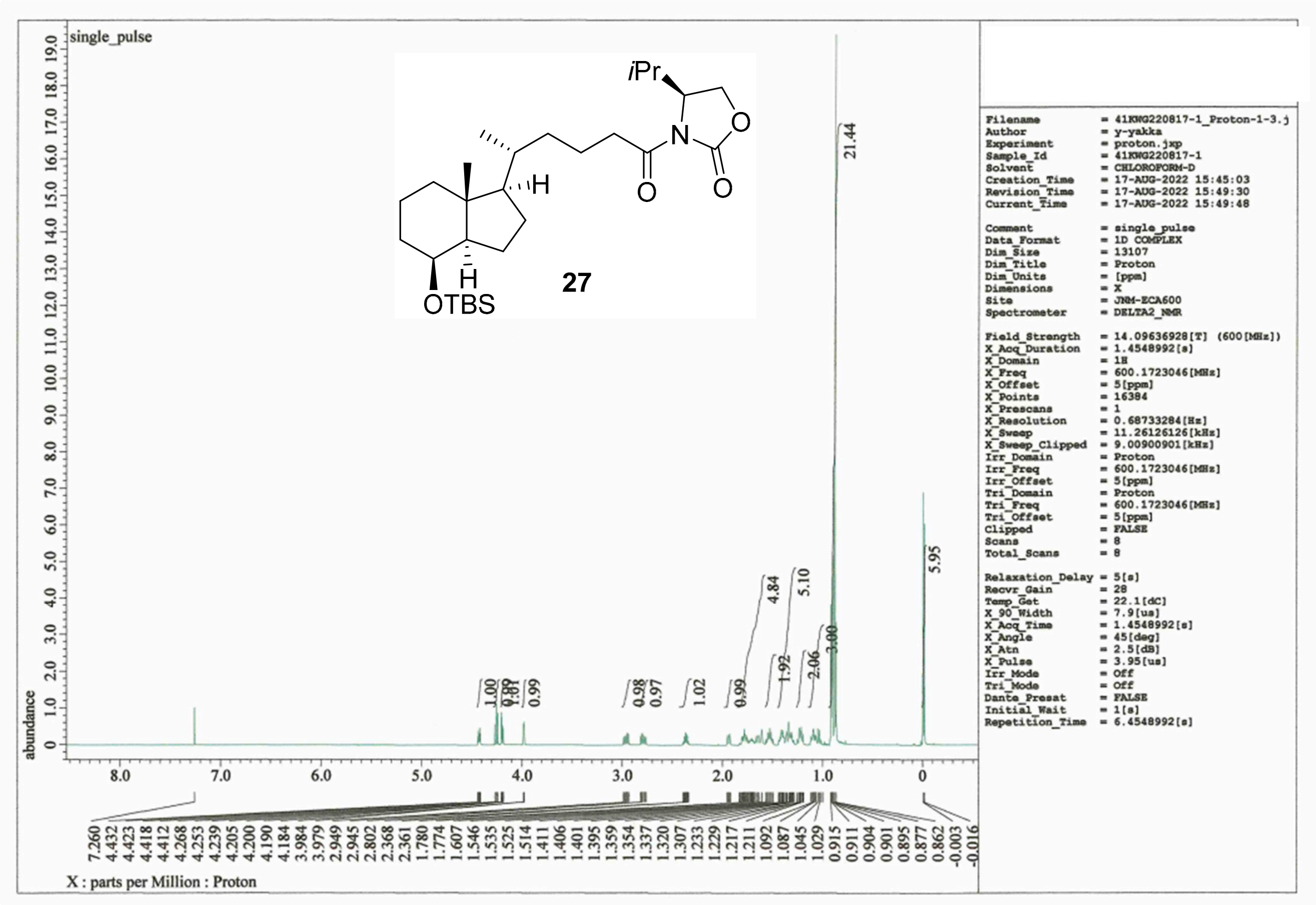
single_pulse
iPr
N
O
O
O
H
H
OTBS
27
21.44
5.95
4.84
5.10
3.00
2.06
1.92
1.00
0.99
0.98
0.97
1.02
0.99
abundance
X : parts per Million : Proton
Filename = 41KWG220817-1_Proton-1-3.j
Author = y-yakka
Experiment = proton.jxp
Sample_Id = 41KWG220817-1
Solvent = CHLOROFORM-D
Creation_Time = 17-AUG-2022 15:45:03
Revision_Time = 17-AUG-2022 15:49:30
Current_Time = 17-AUG-2022 15:49:48
Comment = single_pulse
Data_Format = 1D COMPLEX
Dim_Size = 13107
Dim_Title = Proton
Dim_Units = [ppm]
Dimensions = X
Site = JNM-ECA600
Spectrometer = DELTA2_NMR
Field_Strength = 14.09636928[T] (600[MHz])
X_Acq_Duration = 1.4548992[s]
X_Domain = 1H
X_Freq = 600.1723046[MHz]
X_Offset = 5[ppm]
X_Points = 16384
X_Prescans = 1
X_Resolution = 0.68733284[Hz]
X_Sweep = 11.26126126[kHz]
X_Sweep_Clipped = 9.00900901[kHz]
Irr_Domain = Proton
Irr_Freq = 600.1723046[MHz]
Irr_Offset = 5[ppm]
Tri_Domain = Proton
Tri_Freq = 600.1723046[MHz]
Tri_Offset = 5[ppm]
Clipped = FALSE
Scans = 8
Total_Scans = 8
Relaxation_Delay = 5[s]
Recvr_Gain = 28
Temp_Get = 22.1[dC]
X_90_Width = 7.9[us]
X_Acq_Time = 1.4548992[s]
X_Angle = 45[deg]
X_Atn = 2.5[dB]
X_Pulse = 3.95[us]
Irr_Mode = Off
Tri_Mode = Off
Dante_Presat = FALSE
Initial_Wait = 1[s]
Repetition_Time = 6.4548992[s]

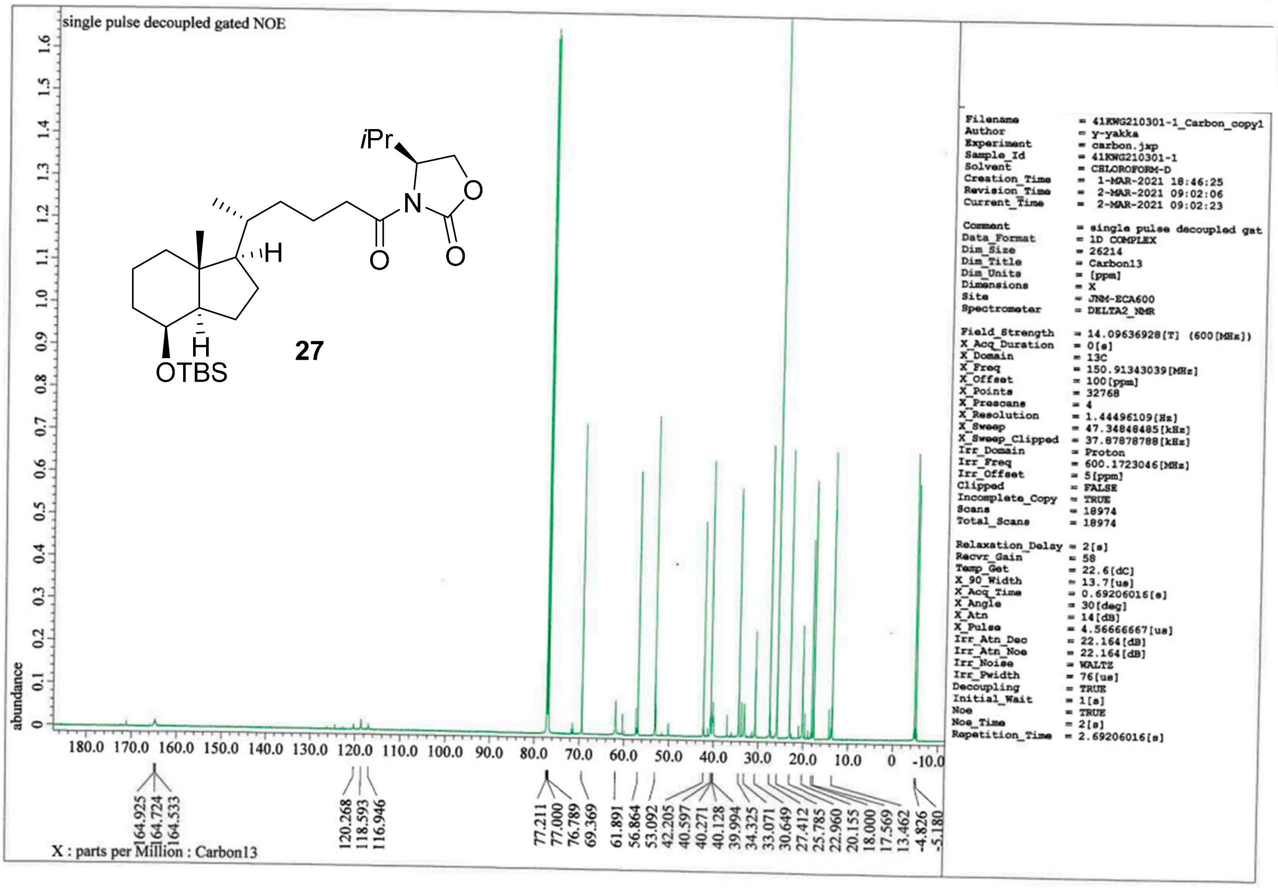
single pulse decoupled gated NOE
iPr
N
O
O
O
H
H
OTBS
27
abundance
X : parts per Million : Carbon13
164.925
164.724
164.533
120.268
118.593
116.946
77.211
77.000
76.789
69.369
61.891
56.864
53.092
42.205
40.597
40.271
40.128
39.994
34.325
33.071
30.649
27.412
25.785
22.960
20.155
18.000
17.569
13.462
-4.826
-5.180
Filename = 41KWG210301-1_Carbon_copy1
Author = y-yakka
Experiment = carbon.jxp
Sample_Id = 41KWG210301-1
Solvent = CHLOROFORM-D
Creation_Time = 1-MAR-2021 18:46:25
Revision_Time = 2-MAR-2021 09:02:06
Current_Time = 2-MAR-2021 09:02:23
Comment = single pulse decoupled gat
Data_Format = 1D COMPLEX
Dim_Size = 26214
Dim_Title = Carbon13
Dim_Units = [ppm]
Dimensions = X
Site = JNM-ECA600
Spectrometer = DELTA2_NMR
Field_Strength = 14.09636928[T] (600[MHz])
X_Acq_Duration = 0[s]
X_Domain = 13C
X_Freq = 150.91343039[MHz]
X_Offset = 100[ppm]
X_Points = 32768
X_Prescans = 4
X_Resolution = 1.44496109[Hz]
X_Sweep = 47.34848485[kHz]
X_Sweep_Clipped = 37.87878788[kHz]
Irr_Domain = Proton
Irr_Freq = 600.1723046[MHz]
Irr_Offset = 5[ppm]
Clipped = FALSE
Incomplete_Copy = TRUE
Scans = 18974
Total_Scans = 18974
Relaxation_Delay = 2[s]
Recvr_Gain = 58
Temp_Get = 22.6[dC]
X_90_Width = 13.7[us]
X_Acq_Time = 0.69206016[s]
X_Angle = 30[deg]
X_Atn = 14[dB]
X_Pulse = 4.56666667[us]
Irr_Atn_Dec = 22.164[dB]
Irr_Atn_Noe = 22.164[dB]
Irr_Noise = WALTZ
Irr_Pwidth = 76[us]
Decoupling = TRUE
Initial_Wait = 1[s]
Noe = TRUE
Noe_Time = 2[s]
Repetition_Time = 2.69206016[s]

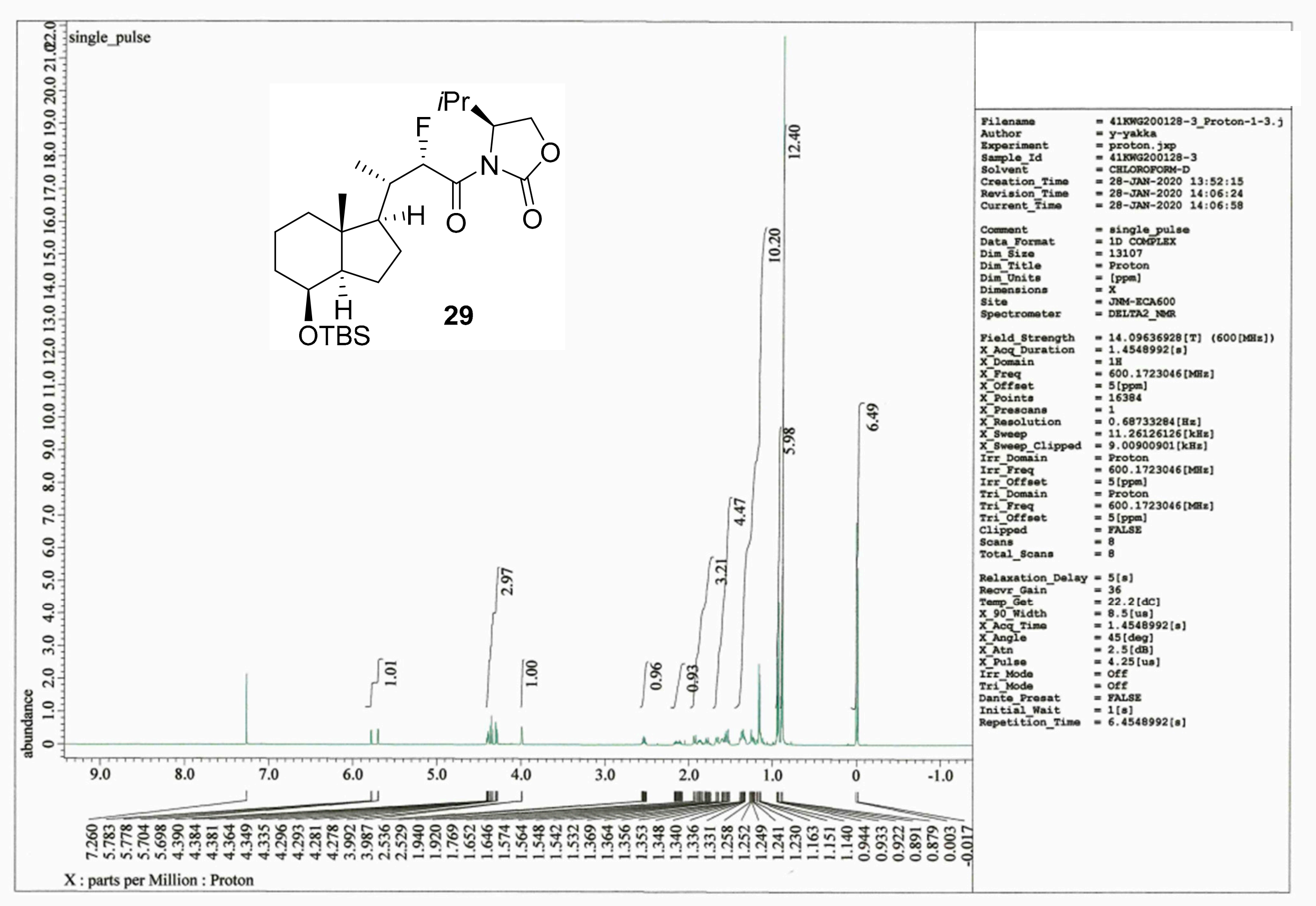
single_pulse
iPr
F
N
O
O
O
H
H
OTBS
29
Filename = 41KWG200128-3_Proton-1-3.j
Author = y-yakka
Experiment = proton.jxp
Sample_Id = 41KWG200128-3
Solvent = CHLOROFORM-D
Creation_Time = 28-JAN-2020 13:52:15
Revision_Time = 28-JAN-2020 14:06:24
Current_Time = 28-JAN-2020 14:06:58
Comment = single_pulse
Data_Format = 1D COMPLEX
Dim_Size = 13107
Dim_Title = Proton
Dim_Units = [ppm]
Dimensions = X
Site = JNM-ECA600
Spectrometer = DELTA2_NMR
Field_Strength = 14.09636928[T] (600[MHz])
X_Acq_Duration = 1.4548992[s]
X_Domain = 1H
X_Freq = 600.1723046[MHz]
X_Offset = 5[ppm]
X_Points = 16384
X_Prescans = 1
X_Resolution = 0.68733284[Hz]
X_Sweep = 11.26126126[kHz]
X_Sweep_Clipped = 9.00900901[kHz]
Irr_Domain = Proton
Irr_Freq = 600.1723046[MHz]
Irr_Offset = 5[ppm]
Tri_Domain = Proton
Tri_Freq = 600.1723046[MHz]
Tri_Offset = 5[ppm]
Clipped = FALSE
Scans = 8
Total_Scans = 8
Relaxation_Delay = 5[s]
Recvr_Gain = 36
Temp_Get = 22.2[dC]
X_90_Width = 8.5[us]
X_Acq_Time = 1.4548992[s]
X_Angle = 45[deg]
X_Atn = 2.5[dB]
X_Pulse = 4.25[us]
Irr_Mode = Off
Tri_Mode = Off
Dante_Presat = FALSE
Initial_Wait = 1[s]
Repetition_Time = 6.4548992[s]
abundance
1.01
2.97
1.00
0.96
0.93
3.21
4.47
10.20
12.40
5.98
6.49
7.260 5.783 5.778 5.704 5.698 4.390 4.384 4.381 4.364 4.349 4.335 4.296 4.293 4.281 4.278 3.992 3.987 2.536 2.529 1.940 1.920 1.769 1.652 1.646 1.574 1.564 1.548 1.542 1.532 1.369 1.364 1.356 1.353 1.348 1.340 1.336 1.331 1.258 1.252 1.249 1.241 1.230 1.163 1.151 1.140 0.944 0.933 0.922 0.891 0.879 0.003 -0.017
X : parts per Million : Proton

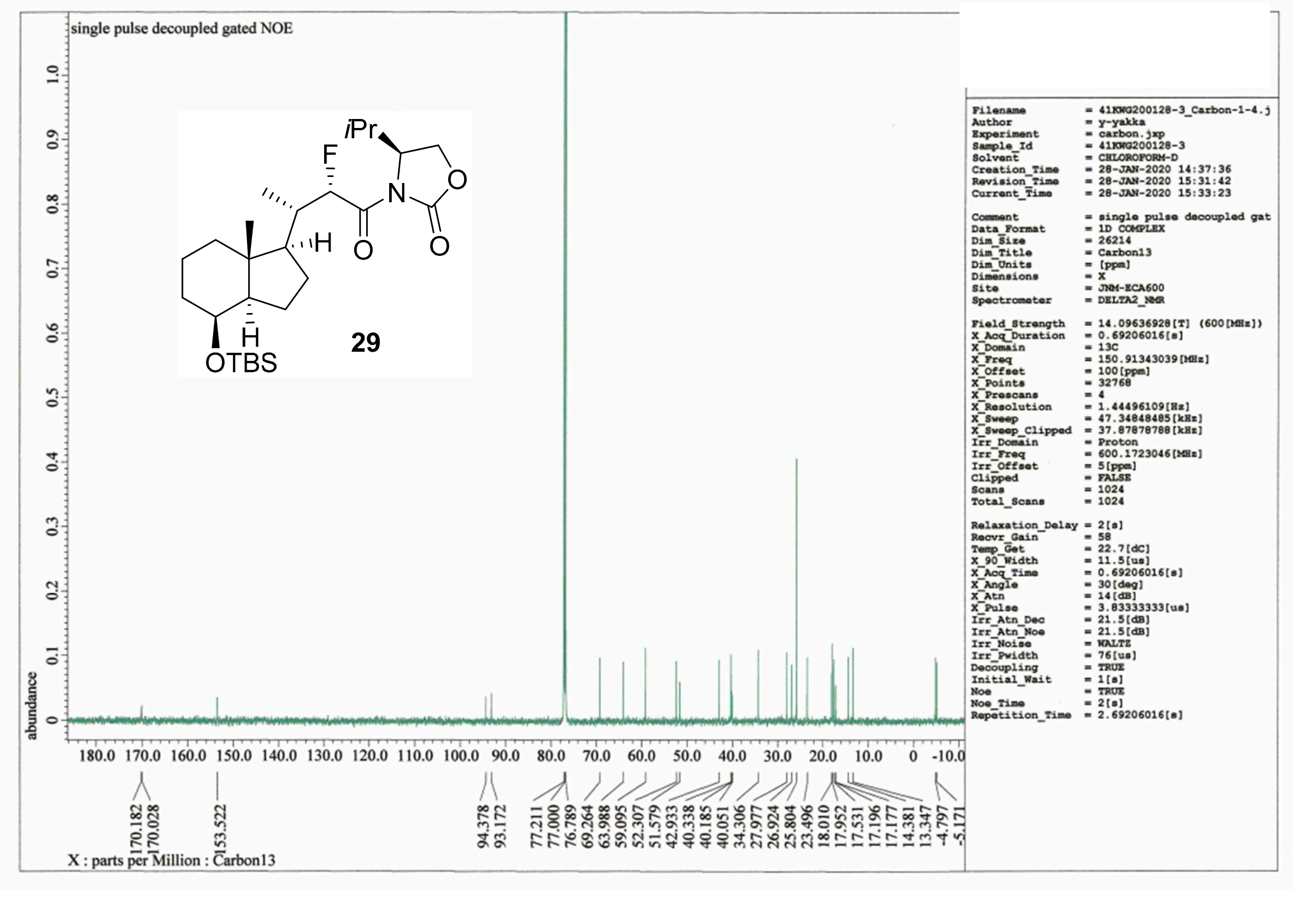
single pulse decoupled gated NOE
iPr
F
N
O
O
O
H
H
OTBS
29
Filename = 41KWG200128-3_Carbon-1-4.j
Author = y-yakka
Experiment = carbon.jxp
Sample_Id = 41KWG200128-3
Solvent = CHLOROFORM-D
Creation_Time = 28-JAN-2020 14:37:36
Revision_Time = 28-JAN-2020 15:31:42
Current_Time = 28-JAN-2020 15:33:23
Comment = single pulse decoupled gat
Data_Format = 1D COMPLEX
Dim_Size = 26214
Dim_Title = Carbon13
Dim_Units = [ppm]
Dimensions = X
Site = JNM-ECA600
Spectrometer = DELTA2_NMR
Field_Strength = 14.09636928[T] (600[MHz])
X_Acq_Duration = 0.69206016[s]
X_Domain = 13C
X_Freq = 150.91343039[MHz]
X_Offset = 100[ppm]
X_Points = 32768
X_Prescans = 4
X_Resolution = 1.44496109[Hz]
X_Sweep = 47.34848485[kHz]
X_Sweep_Clipped = 37.87878788[kHz]
Irr_Domain = Proton
Irr_Freq = 600.1723046[MHz]
Irr_Offset = 5[ppm]
Clipped = FALSE
Scans = 1024
Total_Scans = 1024
Relaxation_Delay = 2[s]
Recvr_Gain = 58
Temp_Get = 22.7[dC]
X_90_Width = 11.5[us]
X_Acq_Time = 0.69206016[s]
X_Angle = 30[deg]
X_Atn = 14[dB]
X_Pulse = 3.83333333[us]
Irr_Atn_Dec = 21.5[dB]
Irr_Atn_Noe = 21.5[dB]
Irr_Noise = WALTZ
Irr_Pwidth = 76[us]
Decoupling = TRUE
Initial_Wait = 1[s]
Noe = TRUE
Noe_Time = 2[s]
Repetition_Time = 2.69206016[s]
abundance
170.182 170.028 153.522 94.378 93.172 77.211 77.000 76.789 69.264 63.988 59.095 52.307 51.579 42.933 40.338 40.185 40.051 34.306 27.977 26.924 25.804 23.496 18.010 17.952 17.531 17.196 17.177 14.381 13.347 -4.797 -5.171
X : parts per Million : Carbon13

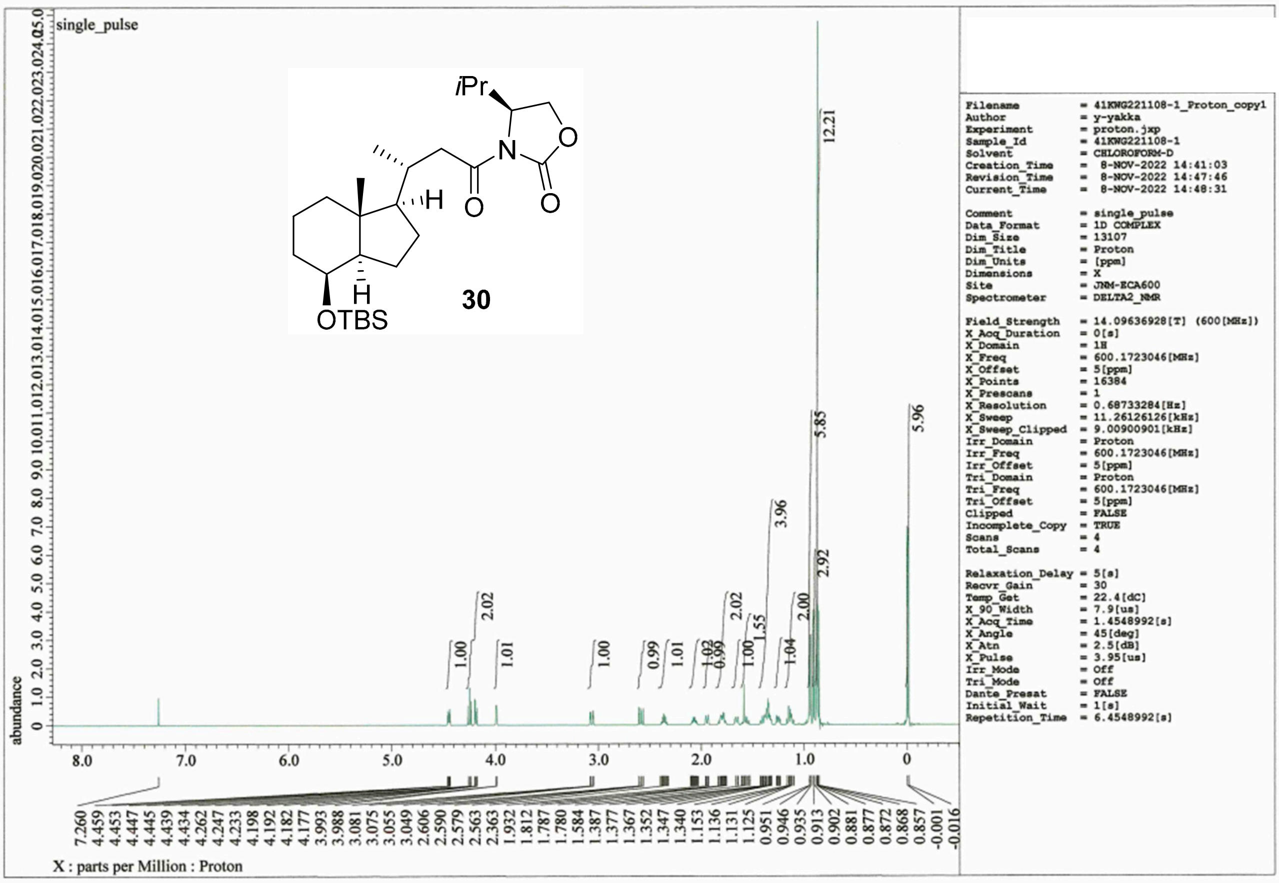
single_pulse
iPr
N
O
O
O
H
H
OTBS
30
12.21
5.85
5.96
3.96
2.92
2.02
2.00
1.55
1.00
1.01
1.00
0.99
1.01
1.02
0.98
1.00
1.04
2.02
abundance
Filename = 41KWG221108-1_Proton_copy1
Author = y-yakka
Experiment = proton.jxp
Sample_Id = 41KWG221108-1
Solvent = CHLOROFORM-D
Creation_Time = 8-NOV-2022 14:41:03
Revision_Time = 8-NOV-2022 14:47:46
Current_Time = 8-NOV-2022 14:48:31
Comment = single_pulse
Data_Format = 1D COMPLEX
Dim_Size = 13107
Dim_Title = Proton
Dim_Units = [ppm]
Dimensions = X
Site = JNM-ECA600
Spectrometer = DELTA2_NMR
Field_Strength = 14.09636928[T] (600[MHz])
X_Acq_Duration = 0[s]
X_Domain = 1H
X_Freq = 600.1723046[MHz]
X_Offset = 5[ppm]
X_Points = 16384
X_Prescans = 1
X_Resolution = 0.68733284[Hz]
X_Sweep = 11.26126126[kHz]
X_Sweep_Clipped = 9.00900901[kHz]
Irr_Domain = Proton
Irr_Freq = 600.1723046[MHz]
Irr_Offset = 5[ppm]
Tri_Domain = Proton
Tri_Freq = 600.1723046[MHz]
Tri_Offset = 5[ppm]
Clipped = FALSE
Incomplete_Copy = TRUE
Scans = 4
Total_Scans = 4
Relaxation_Delay = 5[s]
Recvr_Gain = 30
Temp_Get = 22.4[dC]
X_90_Width = 7.9[us]
X_Acq_Time = 1.4548992[s]
X_Angle = 45[deg]
X_Atn = 2.5[dB]
X_Pulse = 3.95[us]
Irr_Mode = Off
Tri_Mode = Off
Dante_Presat = FALSE
Initial_Wait = 1[s]
Repetition_Time = 6.4548992[s]
8.0 7.0 6.0 5.0 4.0 3.0 2.0 1.0 0
7.260 4.459 4.453 4.447 4.445 4.439 4.434 4.262 4.247 4.233 4.198 4.192 4.182 4.177 3.993 3.988 3.081 3.075 3.055 3.049 2.606 2.590 2.579 2.563 2.363 1.932 1.812 1.787 1.780 1.584 1.387 1.377 1.367 1.352 1.347 1.340 1.153 1.136 1.131 1.125 0.951 0.946 0.935 0.913 0.902 0.881 0.877 0.872 0.868 0.857 -0.001 -0.016
X : parts per Million : Proton

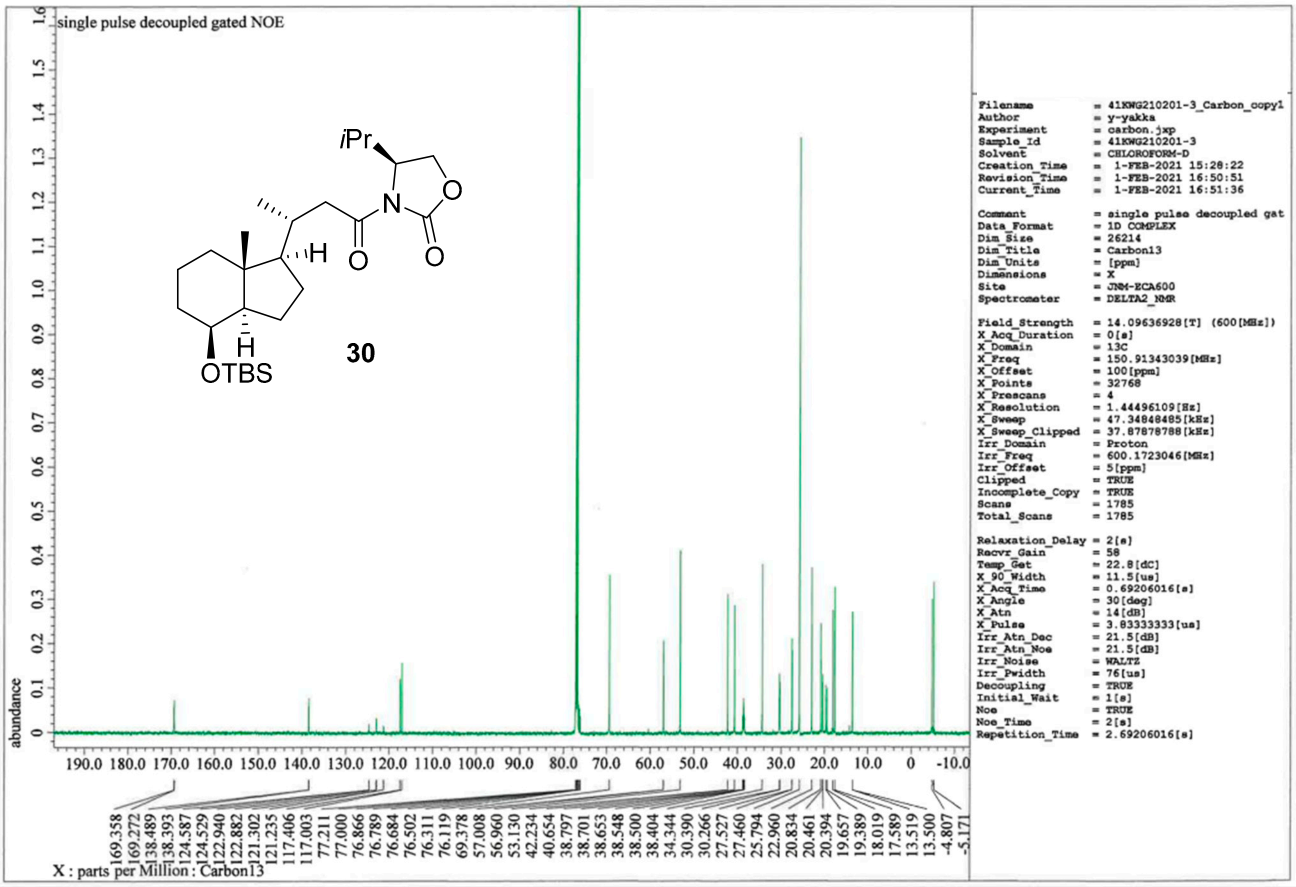
single pulse decoupled gated NOE
iPr
N
O
O
O
H
H
OTBS
30
abundance
Filename = 41KWG210201-3_Carbon_copy1
Author = y-yakka
Experiment = carbon.jxp
Sample_Id = 41KWG210201-3
Solvent = CHLOROFORM-D
Creation_Time = 1-FEB-2021 15:28:22
Revision_Time = 1-FEB-2021 16:50:51
Current_Time = 1-FEB-2021 16:51:36
Comment = single pulse decoupled gat
Data_Format = 1D COMPLEX
Dim_Size = 26214
Dim_Title = Carbon13
Dim_Units = [ppm]
Dimensions = X
Site = JNM-ECA600
Spectrometer = DELTA2_NMR
Field_Strength = 14.09636928[T] (600[MHz])
X_Acq_Duration = 0[s]
X_Domain = 13C
X_Freq = 150.91343039[MHz]
X_Offset = 100[ppm]
X_Points = 32768
X_Prescans = 4
X_Resolution = 1.44496109[Hz]
X_Sweep = 47.34848485[kHz]
X_Sweep_Clipped = 37.87878788[kHz]
Irr_Domain = Proton
Irr_Freq = 600.1723046[MHz]
Irr_Offset = 5[ppm]
Clipped = TRUE
Incomplete_Copy = TRUE
Scans = 1785
Total_Scans = 1785
Relaxation_Delay = 2[s]
Recvr_Gain = 58
Temp_Get = 22.8[dC]
X_90_Width = 11.5[us]
X_Acq_Time = 0.69206016[s]
X_Angle = 30[deg]
X_Atn = 14[dB]
X_Pulse = 3.83333333[us]
Irr_Atn_Dec = 21.5[dB]
Irr_Atn_Noe = 21.5[dB]
Irr_Noise = WALTZ
Irr_Pwidth = 76[us]
Decoupling = TRUE
Initial_Wait = 1[s]
Noe = TRUE
Noe_Time = 2[s]
Repetition_Time = 2.69206016[s]
190.0 180.0 170.0 160.0 150.0 140.0 130.0 120.0 110.0 100.0 90.0 80.0 70.0 60.0 50.0 40.0 30.0 20.0 10.0 0 -10.0
169.358 169.272 138.489 138.393 124.587 124.529 122.940 122.882 121.302 121.235 117.406 117.003 77.211 77.000 76.866 76.789 76.684 76.502 76.311 76.119 69.378 57.008 56.960 53.130 42.254 40.654 38.797 38.701 38.653 38.548 38.500 38.404 34.344 30.390 30.266 27.527 27.460 25.794 22.960 20.834 20.461 20.394 19.657 19.389 18.019 17.589 13.519 13.500 -4.807 -5.171
X : parts per Million : Carbon13

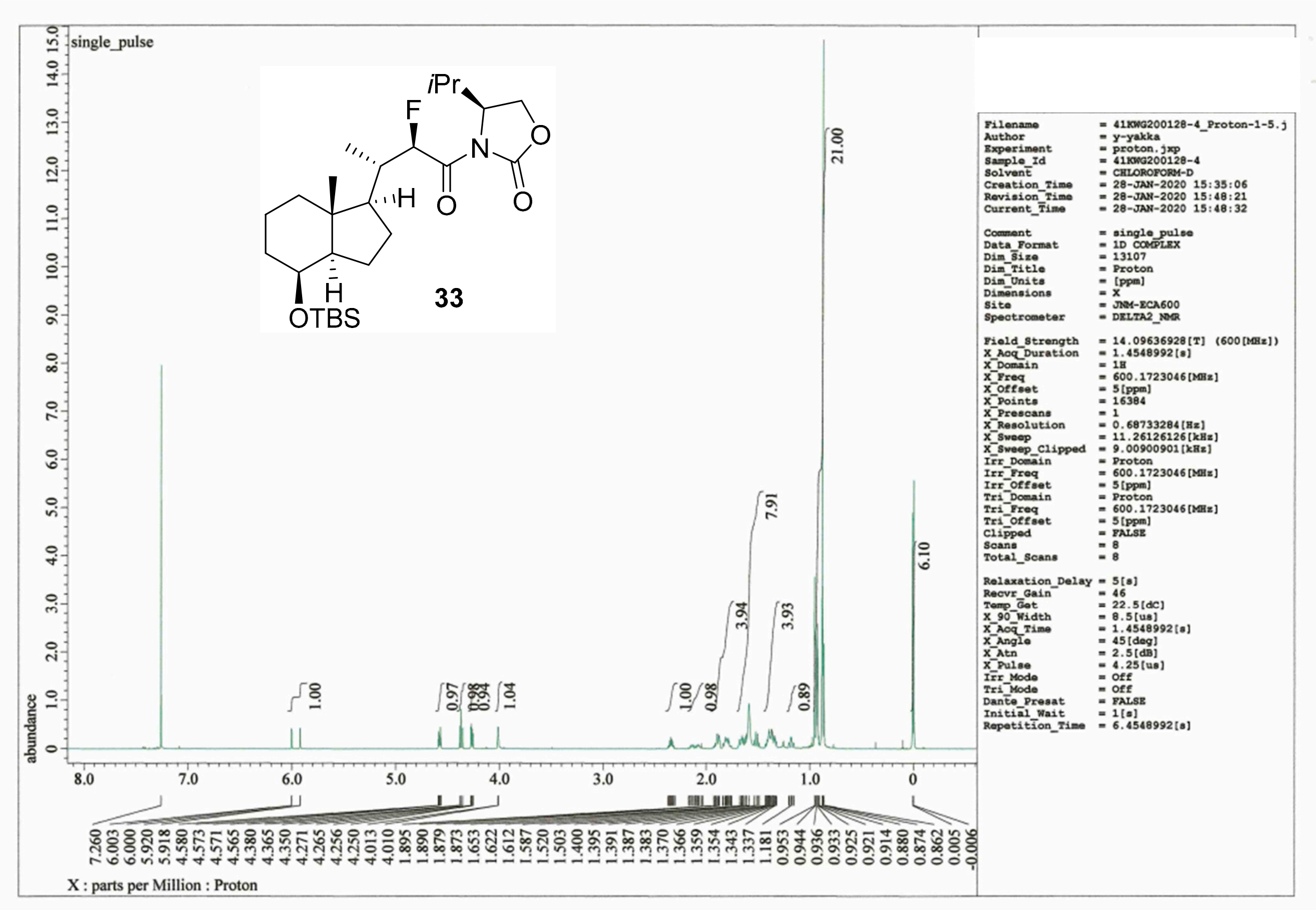
single_pulse
iPr
F
N
O
O
O
H
H
OTBS
33
21.00
7.91
6.10
3.94
3.93
1.00
0.97
0.98
0.94
1.04
1.00
0.98
0.89
abundance
X : parts per Million : Proton
7.260 6.003 6.000 5.920 5.918 4.580 4.573 4.571 4.565 4.380 4.365 4.350 4.271 4.265 4.256 4.250 4.013 4.010 1.895 1.890 1.879 1.873 1.653 1.622 1.612 1.587 1.520 1.503 1.400 1.395 1.391 1.387 1.383 1.370 1.366 1.359 1.354 1.343 1.337 1.181 0.953 0.944 0.936 0.933 0.925 0.921 0.914 0.880 0.874 0.862 0.005 -0.006
Filename = 41KWG200128-4_Proton-1-5.j
Author = y-yakka
Experiment = proton.jxp
Sample_Id = 41KWG200128-4
Solvent = CHLOROFORM-D
Creation_Time = 28-JAN-2020 15:35:06
Revision_Time = 28-JAN-2020 15:48:21
Current_Time = 28-JAN-2020 15:48:32
Comment = single_pulse
Data_Format = 1D COMPLEX
Dim_Size = 13107
Dim_Title = Proton
Dim_Units = [ppm]
Dimensions = X
Site = JNM-ECA600
Spectrometer = DELTA2_NMR
Field_Strength = 14.09636928[T] (600[MHz])
X_Acq_Duration = 1.4548992[s]
X_Domain = 1H
X_Freq = 600.1723046[MHz]
X_Offset = 5[ppm]
X_Points = 16384
X_Prescans = 1
X_Resolution = 0.68733284[Hz]
X_Sweep = 11.26126126[kHz]
X_Sweep_Clipped = 9.00900901[kHz]
Irr_Domain = Proton
Irr_Freq = 600.1723046[MHz]
Irr_Offset = 5[ppm]
Tri_Domain = Proton
Tri_Freq = 600.1723046[MHz]
Tri_Offset = 5[ppm]
Clipped = FALSE
Scans = 8
Total_Scans = 8
Relaxation_Delay = 5[s]
Recvr_Gain = 46
Temp_Get = 22.5[dC]
X_90_Width = 8.5[us]
X_Acq_Time = 1.4548992[s]
X_Angle = 45[deg]
X_Atn = 2.5[dB]
X_Pulse = 4.25[us]
Irr_Mode = Off
Tri_Mode = Off
Dante_Presat = FALSE
Initial_Wait = 1[s]
Repetition_Time = 6.4548992[s]

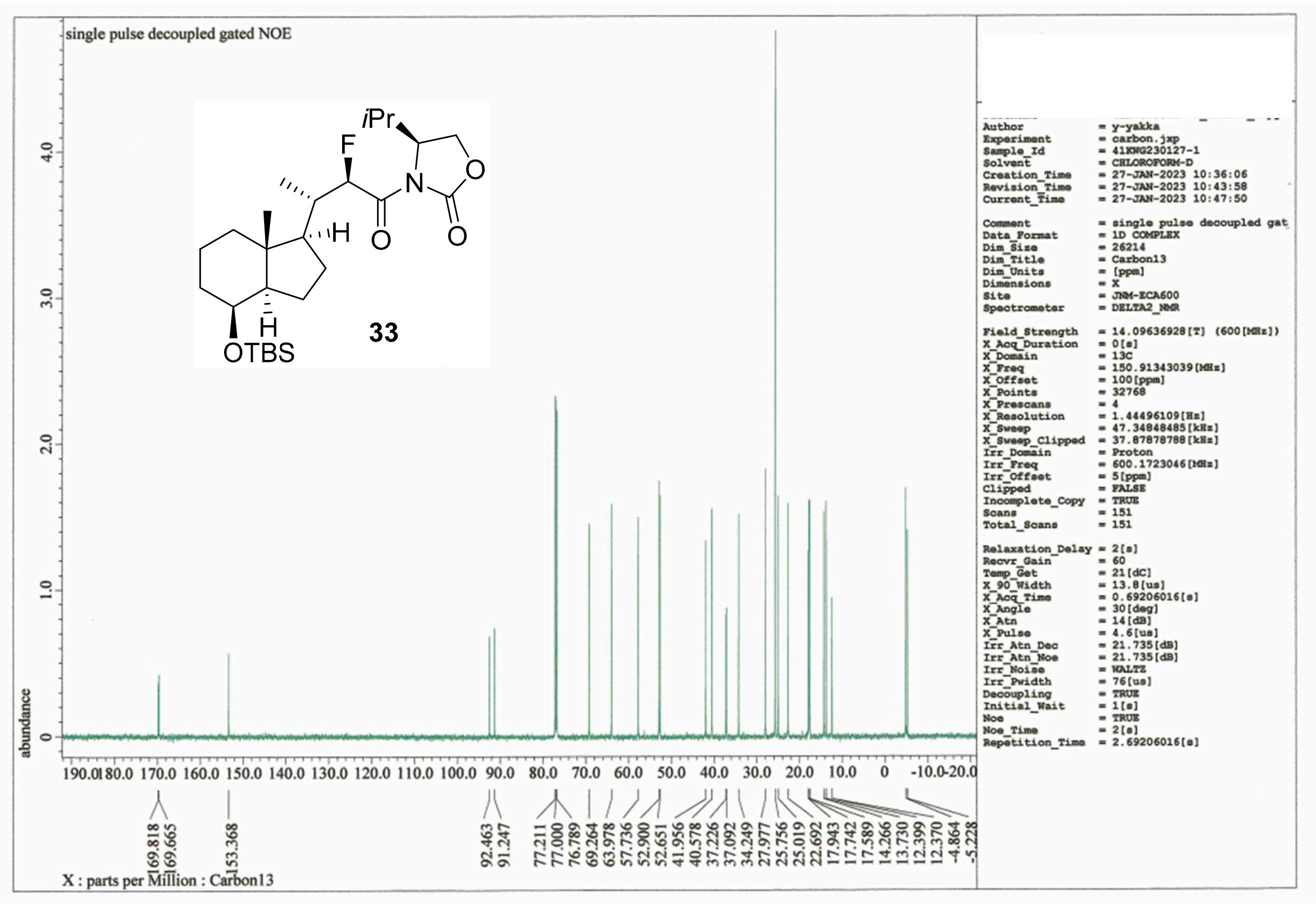
single pulse decoupled gated NOE
iPr
F
N
O
O
O
H
H
OTBS
33
abundance
X : parts per Million : Carbon13
169.818 169.665 153.368 92.463 91.247 77.211 77.000 76.789 69.264 63.978 57.736 52.900 52.651 41.956 40.578 37.226 37.092 34.249 27.977 25.756 25.019 22.692 17.943 17.742 17.589 14.266 13.730 12.399 12.370 -4.864 -5.228
Author = y-yakka
Experiment = carbon.jxp
Sample_Id = 41KWG230127-1
Solvent = CHLOROFORM-D
Creation_Time = 27-JAN-2023 10:36:06
Revision_Time = 27-JAN-2023 10:43:58
Current_Time = 27-JAN-2023 10:47:50
Comment = single pulse decoupled gat
Data_Format = 1D COMPLEX
Dim_Size = 26214
Dim_Title = Carbon13
Dim_Units = [ppm]
Dimensions = X
Site = JNM-ECA600
Spectrometer = DELTA2_NMR
Field_Strength = 14.09636928[T] (600[MHz])
X_Acq_Duration = 0[s]
X_Domain = 13C
X_Freq = 150.91343039[MHz]
X_Offset = 100[ppm]
X_Points = 32768
X_Prescans = 4
X_Resolution = 1.44496109[Hz]
X_Sweep = 47.34848485[kHz]
X_Sweep_Clipped = 37.87878788[kHz]
Irr_Domain = Proton
Irr_Freq = 600.1723046[MHz]
Irr_Offset = 5[ppm]
Clipped = FALSE
Incomplete_Copy = TRUE
Scans = 151
Total_Scans = 151
Relaxation_Delay = 2[s]
Recvr_Gain = 60
Temp_Get = 21[dC]
X_90_Width = 13.8[us]
X_Acq_Time = 0.69206016[s]
X_Angle = 30[deg]
X_Atn = 14[dB]
X_Pulse = 4.6[us]
Irr_Atn_Dec = 21.735[dB]
Irr_Atn_Noe = 21.735[dB]
Irr_Noise = WALTZ
Irr_Pwidth = 76[us]
Decoupling = TRUE
Initial_Wait = 1[s]
Noe = TRUE
Noe_Time = 2[s]
Repetition_Time = 2.69206016[s]
